# Effect of a Machine Learning Recommender System and Viral Peer Marketing Intervention on Smoking Cessation: A Randomized Clinical Trial

**DOI:** 10.1001/jamanetworkopen.2022.50665

**Published:** 2023-01-12

**Authors:** Jamie M. Faro, Jinying Chen, Julie Flahive, Catherine S. Nagawa, Elizabeth A. Orvek, Thomas K. Houston, Jeroan J. Allison, Sharina D. Person, Bridget M. Smith, Amanda C. Blok, Rajani S. Sadasivam

**Affiliations:** 1 Division of Health Informatics and Implementation Science, Department of Population and Quantitative Health Sciences, University of Massachusetts Chan Medical School, Worcester; 2 Wake Forest University School of Medicine, Winston-Salem, North Carolina; 3 Division of Biostatistics and Health Services Research, Population and Quantitative Health Sciences, University of Massachusetts Chan Medical School, Worcester; 4 Spinal Cord Injury Quality Enhancement Research Initiative, Center of Innovation for Complex Chronic Healthcare, Hines VA Medical Center, Chicago, Illinois; 5 Department of Pediatrics and Center for Community Health, Northwestern University Feinberg School of Medicine, Chicago, Illinois; 6 Department of Systems, Populations and Leadership, University of Michigan School of Nursing, Ann Arbor

## Abstract

**Importance:**

Novel data science and marketing methods of smoking-cessation intervention have not been adequately evaluated.

**Objective:**

To compare machine learning recommender (ML recommender) computer tailoring of motivational text messages vs a standard motivational text–based intervention (standard messaging) and a viral peer-recruitment tool kit (viral tool kit) for recruiting friends and family vs no tool kit in a smoking-cessation intervention.

**Design, Setting, and Participants:**

This 2 ×2 factorial randomized clinical trial with partial allocation, conducted between July 2017 and September 2019 within an online tobacco intervention, recruited current smokers aged 18 years and older who spoke English from the US via the internet and peer referral. Data were analyzed from March through May 2022.

**Interventions:**

Participants registering for the online intervention were randomly assigned to the ML recommender or standard messaging groups followed by partially random allocation to access to viral tool kit or no viral tool kit groups. The ML recommender provided ongoing refinement of message selection based on user feedback and comparison with a growing database of other users, while the standard system selected messages based on participant baseline readiness to quit.

**Main Outcomes and Measures:**

Our primary outcome was self-reported 7-day point prevalence smoking cessation at 6 months.

**Results:**

Of 1487 participants who smoked (444 aged 19-34 years [29.9%], 508 aged 35-54 years [34.1%], 535 aged ≥55 years [36.0%]; 1101 [74.0%] females; 189 Black [12.7%] and 1101 White [78.5%]; 106 Hispanic [7.1%]), 741 individuals were randomly assigned to the ML recommender group and 746 individuals to the standard messaging group; viral tool kit access was provided to 745 participants, and 742 participants received no such access. There was no significant difference in 6-month smoking cessation between ML recommender (146 of 412 participants [35.4%] with outcome data) and standard messaging (156 of 389 participants [40.1%] with outcome data) groups (adjusted odds ratio, 0.81; 95% CI, 0.61-1.08). Smoking cessation was significantly higher in viral tool kit (177 of 395 participants [44.8%] with outcome data) vs no viral tool kit (125 of 406 participants [30.8%] with outcome data) groups (adjusted odds ratio, 1.48; 95% CI, 1.11-1.98).

**Conclusions and Relevance:**

In this study, machine learning–based selection did not improve performance compared with standard message selection, while viral marketing did improve cessation outcomes. These results suggest that in addition to increasing dissemination, viral recruitment may have important implications for improving effectiveness of smoking-cessation interventions.

**Trial Registration:**

ClinicalTrials.gov Identifier: NCT03224520

## Key Points

**Question:**

Can a machine learning recommender system and viral peer recruitment improve a digital intervention for smoking cessation?

**Findings:**

In this randomized clinical trial of 1487 people who smoked, those with access to viral peer-recruitment tools had significantly improved smoking cessation outcomes (44.8%) compared with those with no such access (30.8%). There were no differences in cessation outcomes between a machine learning recommender and standard motivational messaging intervention.

**Meaning:**

These findings suggest that viral peer-recruitment tools may improve the dissemination and effectiveness of digital smoking-cessation interventions.

## Teaser

This randomized clinical trial investigates the effect of a machine learning recommender messaging system and viral peer recruitment tool kit on a smoking-cessation intervention.

## Introduction

More than 7 million yearly deaths are attributed to smoking globally, including 480 000 deaths in the US.^1^ In-person smoking-cessation programs have limited reach due to logistical, geographic, and time constraints.^2^ Deciding to use these intensive interventions is a major step for people who smoke given that most such individuals (70%-80%) are not ready to quit.^3^ Smoking-cessation health communication programs accessible via the internet and mobile devices offer a light-touch intervention and fewer barriers to entry. Compared with usual care or attention controls, these programs increase quit rates.^4,5^ Because of this potential, many real-world implementations have been created (eg, the US government SmokeFree website^6^) as alternate resources for people who smoke.^7^ Leaders have called for further innovations to improve the reach and effectiveness of digital interventions to increase their impact.^8^

The promise of big data analytics for behavioral science has been recognized.^9^ However, this promise has not been realized in computer-tailored health communication, a widely used method in health communication programs.^10^ Computer tailoring focuses on selecting the best message for an individual. Standard computer tailoring uses selected variables from patient baseline profiles matched to specific if-then rules to select messages for subsets of patients.^10,11^ Companies like Amazon use a special class of algorithms (recommender systems based on artificial intelligence and machine learning) to select messages for individuals.^12,13^ Recommender systems can be programmed to adapt to ongoing individual and group user feedback to improve tailoring over time. We developed a similar machine learning–based recommender system for computer tailoring, and our pilot studies demonstrated modest improvement in smoking cessation rates.^14,15,16^

Currently, viral peer marketing (ie, providing participants with a tool kit to recruit friends or family members) is a preferred method for finding new research participants for online studies given that potential new recruits are more likely to trust a referral from a friend.^17^ In our prior pilot study, we found that in approximately 1 year (July 2013 to September 2014), viral peer recruitment was associated with a 4-fold increase in the number of individuals in our sample using the digital tobacco intervention.^18^ Many participants noted that having the opportunity and tools to recruit peers was beneficial to their own quit smoking efforts (75.4%) and motivated them to get support from those around them.^18^

Our pilot data suggested that viral peer marketing and recommender system functions could synergistically affect each other to improve the reach and effectiveness of our intervention. Although the primary function of the viral peer marketing (viral tool kit) is to increase the reach, it may lead to increased smoking cessation, with the peer recruiter serving as a positive role model to those recruited.^19,20^ In addition to promoting cessation, the recommender system (ML recommender) may lead to more engaged participants (due to the increased relevance of the messages), potentially motivating them to use the viral marketing tools, increasing the reach of the digital intervention. Thus, we designed our hybrid dissemination and effectiveness trial as a 2 × 2 factorial randomized large trial to test the viral tool kit and ML recommender independently and their interaction effect on reach and smoking cessation.^21^

## Methods

This randomized clinical trial is reported in accordance with the Consolidated Standards of Reporting Trials (CONSORT) reporting guideline and template for intervention description and replication checklist. The study was approved by the University of Massachusetts Chan Medical School Institutional Review Board, and participants provided informed consent online.

### Study Design

Our protocol for this study is described elsewhere.^21^ Briefly, all participants registering on the digital tobacco intervention site between August 2017 and March 2019 were first randomly assigned to ML recommender or standard messaging groups. They were then allocated to receive the viral tool kit based on their recruitment source, using a partial randomization approach. Participants recruited directly to the study (via search engine advertisements and ResearchMatch, a free and secure online tool developed by Vanderbilt University)^22^ were randomized to receive or not receive the viral tool kit, while those who self-reported recruitment by their peers were assigned to receive access to the viral tool kit. Our respective comparisons were a standard motivational messaging intervention (standard messaging) and no access to the viral peer marketing tool kit (no viral tool kit). This study presents 3 hypotheses related to our smoking-cessation outcomes. In the first hypothesis (H1), we tested whether quit rates among individuals exposed to the fully enhanced group (ML recommender and viral tool kit) were greater than those assigned to the other 3 groups: ML recommender and no tool kit, standard messaging and viral tool kit, and standard messaging and no tool kit. Our second hypothesis (H2) compared quit rates between individuals who were exposed to the viral tool kit with any type of messaging intervention. The third hypothesis (H3) compared quit rates of the ML recommender and standard messaging groups with any type of viral peer-recruitment tool set access. These hypotheses were tested within the Decide2Quit (University of Massachusetts)^23,24^ digital tobacco intervention that included self-management functions, online community, and peer support accessed through the website interface. The trial protocol and statistical analysis plan are provided in Supplement 1.

### Participants and Recruitment

Participants were current smokers aged 18 years and older who spoke English. Participants were recruited from the US online (via Google and Facebook), via SmokeFree^6^ and ResearchMatch, and using peer recruitment.^25^ To recruit online, we developed and posted online advertisements customized to appear to smokers searching for quit smoking–related search terms online. We posted a summary of our project with contact information on the SmokeFree website’s “Join a Research Study” webpage. Volunteers on ResearchMatch were contacted by our research team if they matched our eligibility criteria and agreed to be contacted. Lastly, we tested peer recruitment (access to the viral tool kit) for increasing access to the digital tobacco intervention site as a recruitment source.

### Randomization

We randomized participants using a table conducted in random blocks of different sizes (8 and 12 participants) to ensure balance among groups and reduce predictability of the allocation process. Randomization occurred in 2 stages at the time of initial registration. Participants were first randomized to receive messages from the ML recommender or standard messaging communication systems. This was followed by an allocation to receive or not receive access to the viral tool kit depending on the recruitment source captured via a self-report question presented to participants immediately after they consented to the study. The question was “How did you hear about Decide2Quit?” and answer choices included a friend or family member, online ad (Facebook or Google), ResearchMatch, or the SmokeFree government site. All peer-recruited participants (those who indicated that they were referred by friends or family) received access to the viral tool kit to reduce cross-contamination given that these peer-recruited participants could communicate with their peers about the viral tool kit. Participants who indicated other sources were randomly allocated to receive or not receive the viral tool kit. Study staff were blinded to allocation during initial baseline assessment and follow-up.

### Interventions

#### Computer-Tailored Health Communication Systems

Participants received 2 email messages weekly in the first 4 weeks after registration. They then received 1 email message per week for the remaining 4 months until they reached 6 months from their registration date. Because our goal was to evaluate the selection method, both systems selected from the same database of messages. The messaging database included messages that were developed in a prior study^26^ and written by experts (eg, behaviorists, physicians, and nurses) and peers, informed by current guidelines and social cognitive theory.^3,27^ However, the ML recommender and standard messaging systems differed in how messages were selected for participants. The standard messaging system selected messages for participants based on their baseline readiness to quit (described subsequently).^10,11^ The ML recommender system used feedback from participants to improve message selection. In this study, we used participant feedback from prior data collection from 900 then-current or former smokers and participants in this study.^28,29^

#### Access to Viral Peer-Recruitment Tool Kit

We provided peer-recruitment tools, including a social media website plug-in that allowed participants to send private recruitment messages to friends or family and email referrals.^18^ We encouraged users to recruit friends or family members who were smokers during registration. We also sent 1 weekly motivational email encouraging peer recruitment throughout the study period. Participants additionally had access to an online training video and a recruitment tracker. However, participants were allowed to recruit without using our tools given that our goal was to increase recruitment of participants via peer recruitment. Unlike some other studies,^30,31^ we did not incentivize participants to recruit others to the study in an effort to mimic real-world settings.

### Data Collection

Participants received $25 incentives at each data collection time (baseline registration, 1 week, 1 month, and 6 months). We collected self-reported baseline data and sociodemographic information online as a 2-part survey (at registration and 1 week) to reduce participant burden. We sent up to 4 email reminders over 2 weeks to complete the surveys online. If participants failed to respond to our email messages for the 6-month survey, we contacted them to complete the survey over the phone. Data collection was completed in September 2019.

### Measures

At baseline, participants self-reported their age category, sex, race, ethnicity, education level (high school or less, some college or technical school, or college graduate), financial status (difficulty paying for medical care), and current number of cigarettes smoked per day. Given that there are known differences in smoking status and cessation by racial and ethnic background and because we analyzed at these levels in other studies, we collected race and ethnicity data for this study. Available categories for race were African American, American Indian or Alaska Native, Asian, Native Hawaiian or other Pacific Islander, and White; we combined American Indian or Alaska Native, Asian, and Native Hawaiian or other Pacific Islander as other race due to small sample sizes. For ethnicity, participants could self-report as Hispanic or Latino or not Hispanic or Latino. We assessed readiness to quit smoking by asking, “People often go through different steps when quitting smoking. Where are you in the process?” (available responses were “I’m not thinking about quitting,” “I set a quit date,” and “I quit today or I’ve already quit”). Our primary outcome was 6-month 7-day point prevalence abstinence assessed using the question “Do you currently smoke cigarettes (smoked even 1 puff in the last 7 days)?”^32^ This self-report outcome and time point are valid, have been commonly used in other cessation trials,^33,34,35^ and are acceptable to researchers.^36^ For participants who self-reported quitting, we offered an additional $50 incentive if they were willing to submit a NicAlert nicotine saliva test (Nymox).^37,38,39^ For participants willing to do so, we mailed strips with clear instructions and a link to an online location for participants to upload images of their results. The nicotine saliva test is a semiquantitative method that uses a dipstick to measure the level of cotinine in a sample of saliva. The results were read as 0 to 6, and as recommended, any value of 1 or greater was considered to indicate tobacco use.^40,41,42^ A secondary outcome of risk reduction was assessed as the difference between the number of cigarettes smoked per day at 6-month follow-up compared with baseline.

### Sample Size

Assuming a comparison cessation rate of 15%^43^ and a 2-sided significance level of 0.05, a sample size of 300 participants in each group would achieve 80% power to detect a difference of 9% in quit rates between any 2 groups (quit rate in intervention = 24%) based on a *Z*-test with pooled variance. Therefore, the targeted sample size was 300 participants for each of 4 allocation groups (Figure).

**Figure.**
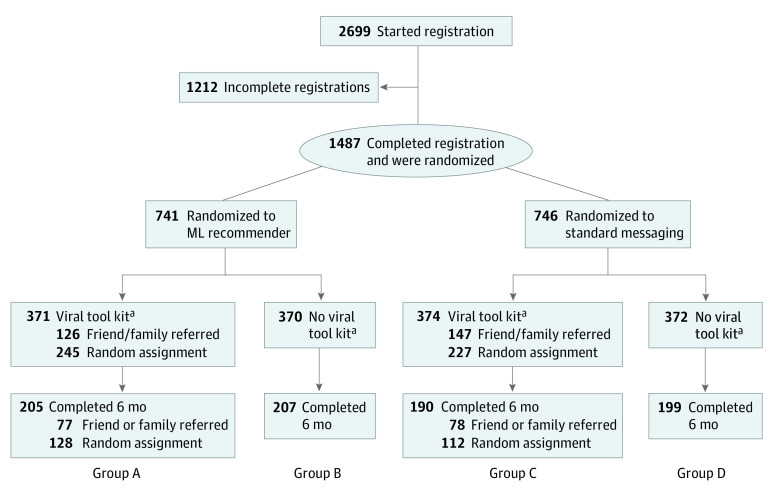
Study Flowchart ML recommender indicates machine learning recommender computer-tailored motivational texting intervention; standard messaging, standard motivational texting intervention; viral tool kit, viral peer-recruitment tool kit. Group A was the fully enhanced group, exposed to the ML recommender and viral tool kit. Group B was exposed to the ML recommender with no viral tool kit. Group C was exposed to standard messaging with viral tool kit. Group D was exposed to standard messaging with no viral tool kit. ^a^Partial randomization: individuals referred by family or friends were allocated to the viral tool kit group, while those recruited from other sources were randomized.

### Statistical Analysis

Statistical analyses were performed using Stata/IC statistical software version 15.1 (StataCorp). For cessation analysis, we used the 7-day point prevalence cessation that was self-reported by participants at 6 months as the outcome variable and used logistic regression and intent-to-treat analysis (ie, participants were analyzed according to their original study groups assigned by randomization). The 3 subhypotheses of H1 were analyzed using logistic regression for a 2 × 2 factorial design with an interaction term. We analyzed H2 and H3 by testing with indicator variables for combined group membership, as described subsequently in more detail. We created unadjusted models and models that were adjusted for sex and education. Each hypothesis was examined by a complete case analysis (primary analysis) and 2 sensitivity analyses: penalty imputation, in which participants with missing outcomes were regarded as smoking, and multiple imputation of missing outcomes. For complete case and penalty imputation analyses, we report unadjusted and adjusted regression results accounting for unbalanced covariates introduced by peer recruitment. For multiple imputation, we stratified the imputation of missing outcome values by study groups and generated 100 imputation sets. The imputation model built on covariates that were unbalanced due to missing data or predictive for outcome values. The same covariates were used to adjust the analysis model.

H1 comprised 3 subhypotheses. The first H1 subhypothesis compared participants exposed to the ML recommender and viral tool kit interventions (group A) vs those exposed only to the ML recommender intervention (group B). The second H1 subhypothesis compared participants in group A vs those who were exposed only to the viral tool kit and standard messaging (group C). The third H1 subhypothesis compared individuals in group A vs those who were exposed to standard messaging but not the viral tool kit (group D) (Figure).

H2 compared 2 combined groups (individuals exposed to the ML recommender intervention regardless of exposure to the viral tool kit [A and B]) vs all others (C and D). This hypothesis also compared 2 additional combined groups (individuals exposed to the viral tool kit regardless of exposure to the ML recommender [A and C]) vs all others (B and D) (Figure).

Although the number of nicotine tests was small, we examined test results for all hypotheses by categorizing tests by smoker vs nonsmoker groups and used the χ^2^ statistic to test for differences. We also tested all 3 hypotheses with risk reduction analysis as our outcome. We limited the maximum number of cigarettes smoked per day at baseline to 75 cigarettes given that there were 3 outliers reporting more than 100 cigarettes per day. If individuals reported not smoking at 6 months, we set their number of cigarettes per day at 6 months to 0. We used a *t* test to compare the difference in mean risk reduction between study groups. Risk reduction analysis was conducted only on complete case data. A 2-sided *P* < .05 was considered statistically significant. Data were analyzed from March through May 2022.

## Results

Among 1487 participants (444 aged 19-34 years [29.9%], 508 aged 35-54 years [34.1%], 535 aged ≥55 years [36.0%]; 1101 [74.0%] females; 189 Black [12.7%] and 1101 White [78.5%]; 106 Hispanic [7.1%]) (Table 1), 741 individuals were randomly assigned to the ML recommender group and 746 participants to the standard messaging group (Figure). Viral tool kit access was provided to 745 participants, and 742 participants received no such access. Among all randomized individuals, 801 participants completed their 6-month survey and 686 participants did not, for a retention rate of 53.4% and a lost to follow-up rate of 46.1%. We found significant differences at baseline in sex or education across groups.

**Table 1.** Baseline Participant Characteristics by Study Group

Characteristic	Participants, No. (%) (N = 1487)^a^			
	Group A (n = 371)	Group B (n = 370)	Group C (n = 374)	Group D (n = 372)
Age group, y				
19-34	109 (29.4)	98 (26.5)	127 (34.0)	110 (29.6)
35-54	129 (34.8)	132 (35.7)	130 (34.8)	117 (31.5)
≥55	133 (35.9)	140 (37.8)	117 (31.3)	145 (39.0)
Sex				
Female	259 (69.8)	281 (76.0)	270 (72.2)	291 (78.2)
Male	112 (30.2)	89 (24.1)	104 (27.8)	81 (21.8)
Race				
African American or Black	49 (13.2)	49 (13.2)	47 (12.6)	44 (11.8)
White	299 (80.6)	298 (80.5)	294 (78.6)	300 (80.7)
Other^b^	23 (6.2)	23 (6.2)	33 (8.8)	28 (7.5)
Ethnicity				
Total with data, No.	354	349	347	347
Hispanic or Latino	22 (6.2)	22 (6.3)	27 (7.8)	35 (10.1)
Not Hispanic or Latino	332 (93.8)	327 (93.7)	320 (92.2)	312 (89.9)
Education level				
Total with data, No.	245	257	246	239
≤High school	73 (29.8)	69 (26.9)	95 (38.6)	62 (25.9)
Some college or technical school	108 (44.1)	111 (43.2)	98 (39.8)	109 (45.6)
College graduate	64 (26.1)	77 (30.0)	53 (22.0)	68 (28.0)
Difficulty paying for medical care				
Total with data, No.	246	257	249	239
Very hard or hard	90 (36.6)	83 (32.3)	82 (32.9)	75 (31.4)
Somewhat hard	77 (31.3)	86 (33.5)	88 (35.3)	99 (41.4)
Not very hard	79 (32.1)	88 (34.2)	79 (31.7)	65 (27.2)
Readiness to quit smoking				
Total with data, No.	368	365	372	371
I am not thinking about quitting	17 (4.6)	10 (2.7)	17 (4.6)	18 (4.9)
I am thinking of quitting, or I have set a quit date	303 (82.3)	299 (81.9)	294 (79.0)	286 (77.1)
I quit today or have already quit	49 (13.0)	56 (15.3)	61 (16.4)	67 (18.1)
Cigarettes smoked, No./d^c^				
0-5	53 (14.3)	39 (10.5)	55 (14.8)	48 (12.9)
6-20	253 (68.2)	248 (67.0)	246 (66.0)	238 (64.0)
≥21	65 (17.5)	83 (22.4)	72 (19.3)	86 (23.1)

^a^ Group A was the fully enhanced group, exposed to the machine learning recommender computer-tailored motivational texting intervention (ML recommender) and viral peer-recruitment tool kit (viral tool kit). Group B was exposed to the ML recommender with no viral tool kit. Group C was exposed to the standard motivational texting intervention (standard messaging) with viral tool kit. Group D was exposed to standard messaging with no viral tool kit.

^b^ American Indian or Alaska Native, Asian, and Native Hawaiian or other Pacific Islander individuals were combined as other race due to low sample sizes.

^c^ Maximum number of baseline cigarettes per day was set at 75 cigarettes given that there were 3 outliers reporting more than 100 cigarettes/d.

In H1, the fully enhanced group (A) had significantly increased quit rates compared with the group exposed to the ML recommender with no access to the viral tool kit (B) (89 of 205 participants [43.4%] vs 57 of 207 participants [27.5%] with outcome data; adjusted odds ratio [aOR], 1.91; 95% CI, 1.20-3.03) (Table 2). In H2, we found significant baseline differences between individuals exposed to the viral tool kit (groups B and D) and those not receiving the viral tool kit (groups A and C) in sex (529 [71.0%] female vs 572 [77.1%] female; *P* = .007) and education level (eg, 117 college graduates [23.8%] vs 145 college graduates [29.2%]; *P* = .02) (eTable 1 in Supplement 2). We additionally found that individuals with access to the viral tool kit (groups A and C) had significant improvements in the 6-month 7-day point prevalence smoking-cessation outcome compared with those without the viral tool kit (groups B and D) (177 of 395 participants [44.8%] vs 125 of 406 participants [30.8%] with outcome data; aOR, 1.48; 95% CI, 1.11-1.98) (Table 2). There was a significant decrease in the mean (SD) number of cigarettes smoked per day between these groups (−10.2 [10.8] cigarettes/d vs −8.6 [10.8] cigarettes/d; *P* = .04).

**Table 2.** Smoking Cessation Rates at 6 mo

Group comparison^a^	Participants, No./total No. (%) (n = 801)^b^	Unadjusted model		Model adjusted for covariates^c^	
		OR (95% CI)	*P* value	OR (95% CI)	*P* value
**Hypothesis 1**					
Subhypothesis 1					
Group A	89/205 (43.4)	2.02 (1.34-3.05)	.001	1.91 (1.20-3.03)	.005
Group B	57/207 (27.5)	1 [Reference]		1 [Reference]	
Subhypothesis 2					
Group A	89/205 (43.4)	0.89 (0.60-1.32)	.56	0.82 (0.52-1.28)	.39
Group C	88/190 (46.3)	1 [Reference]		1 [Reference]	
Subhypothesis 3					
Group A	89/205 (43.4)	1.48 (0.99-2.21)	.06	1.24 (0.79-1.95)	.41
Group D	68/199 (34.2)	1 [Reference]		1 [Reference]	
**Hypothesis 2**					
Groups A and C (viral tool kit)	177/395 (44.8)	1.54 (1.19-1.99)	.001	1.48 (1.11-1.98)	.01
Groups B and D (no viral tool kit)	125/406 (30.8)	1 [Reference]		1 [Reference]	
**Hypothesis 3**					
Groups A and B (ML recommender)	146/412 (35.4)	0.93 (0.71-1.20)	.56	0.81 (0.61-1.08)	.16
Groups C and D (standard messaging)	156/389 (40.1)	1 [Reference]		1 [Reference]	

Abbreviations: ML recommender, machine learning recommender computer-tailored motivational texting intervention; OR, odds ratio; standard messaging, standard motivational texting intervention; viral tool kit, viral peer-recruitment tool kit.

^a^ Group A was the fully enhanced group, exposed to the ML recommender and viral tool kit. Group B was exposed to the ML recommender with no viral tool kit. Group C was exposed to standard messaging with viral tool kit. Group D was exposed to standard messaging with no viral tool kit.

^b^ Analyses are among participants with complete data at 6 months.

^c^ Multivariable logistic regression was adjusted for sex and education level.

In H3, there were no baseline differences between individuals exposed to the ML recommender (groups A and B) and those exposed to standard messaging (groups C and D) (eTable 2 in Supplement 2). There was no significant difference in 6-month smoking cessation between ML recommender (146 of 412 participants [35.4%] with outcome data) and standard messaging (156 of 389 participants [40.1%] with outcome data) groups (aOR, 0.81; 95% CI, 0.61-1.08) (Table 2) or mean (SD) decrease in cigarettes smoked per day (−8.9 [10.5] cigarettes/d vs −9.9 [10.8] cigarettes/d; *P* = .20).

In 2 sensitivity analyses (penalty and multiple imputation) on 6-month smoking cessation, results were consistent with the complete case analysis for all hypotheses (eTables 3 and 4 in Supplement 2). For example, there was no significant difference in 6-month smoking cessation between the ML recommender (146 participants [19.7%]) and standard messaging (156 participants [20.9%]) groups when treating missing outcomes as smoking (eTable 3 in Supplement 2). Our secondary analysis compared smoking cessation and decrease in number of cigarettes smoked per day at 6 months by self-reported referral source. Among 155 participants with complete data who self-reported being recruited by friends or family members, 94 participants quit smoking (60.6%) compared with 83 of 240 participants with complete data who were recruited from other sources (34.6%; aOR, 2.56; 95% CI, 1.66-3.96) (Table 3). Participants who self-reported being recruited by friends or family members had a mean (SD) decrease of −12.1 (9.4) cigarettes/d, which was significantly different from the mean (SD) decrease of −8.7 (10.9) cigarettes/d in those who were recruited from other sources (*P* < .001).

**Table 3.** Sensitivity Analysis of Fully Randomized Group vs Groups Partially Allocated With or Without Viral Tool Kit

Group^a^	Randomization or allocation	No./total No. (%) (N = 1487)	Unadjusted model		Model adjusted for covariates ^b^	
			OR (95% CI)	*P* value	OR (95% CI)	*P* value
Viral tool kit groups (A and C)	Fully randomized participants^c^	83/240 (34.6)	1.19 (0.85-1.67)	.32	1.26 (0.86-1.86)	.23
	Allocated participants^d^	94/155 (60.6)	3.46 (2.36-5.09)	<.001	2.56 (1.66-3.96)	<.001
No viral tool kit groups (B and D)	All participants fully randomized	125/406 (30.8)	1 [Reference]	NA	1 [Reference]	NA

Abbreviations: ML recommender, machine learning recommender computer-tailored motivational texting intervention; NA, not applicable; OR, odds ratio; standard messaging, standard motivational texting intervention; viral tool kit, viral peer-recruitment tool kit.

^a^ Group A was the fully enhanced group, exposed to the ML recommender and viral tool kit. Group B was exposed to the ML recommender with no viral tool kit. Group C was exposed to standard messaging with viral tool kit. Group D was exposed to standard messaging with no viral tool kit.

^b^ Multivariable logistic regression, adjusted for age, sex, and education level reported through the baseline survey, was used for analyses for the complete study sample and study sample with smoking data missing. The analysis model for multiple imputation was adjusted by age, race, sex, education level, and smoking status and number of cigarettes per day measured at baseline.

^c^ Self-reported as recruited by methods other than the viral peer recruitment, including social media ads, web browser ads, ResearchMatch, and the government SmokeFree website.^25^

^d^ Self-reported as recruited via viral peer recruitment.

## Discussion

This randomized clinical trial examined 6-month smoking-cessation data among 1487 people enrolled in a digital smoking-cessation trial. Much of the effect in smoking cessation in our study may have been due to access to the viral peer recruitment tool kit, as our H2 results suggested. Viral peer recruitment draws strength from the constructs of relatedness as proposed in self-determination theory.^44^ Relatedness among individuals, or the desire to feel connected to others, can support behavior change and may explain the significant difference in H2 results.^44,45^ For the recruiter, the act of recruiting others may have promoted accountability to act as a role model and hence promoted cessation. In our pilot study,^18^ many participants reported that the act of peer recruitment motivated them to quit smoking. Conversely, being recruited by a family or peer may also motivate the individual to quit smoking (because the individual does not want to disappoint the friend or family member). Our secondary analyses for this hypothesis revealed that participants who self-reported that they were peer-recruited were more likely to quit than those who self-reported that they were recruited by other means. When individuals engage in activities that are social in nature, such as providing social support, perceptions of relatedness play an important role in motivation and increasing engagement.^46^

There could be several reasons for the H3 null finding. We were comparing the recommender system against a robust standard messaging system that was proven effective in a prior trial.^15^ These systems selected messages from the same database; thus, the only difference was the method of message selection. Our recommender system was programmed with minimal data from 900 participants, while the recommender systems used by large companies have been programmed on large amounts of user data, and this difference may have had an effect on our outcomes.

### Limitations

This study has several limitations. We did not adjust for multiple comparisons^47^ given that our hypotheses were planned before data collection and were supported by robust pilot data, thus lessening the probability of false positive findings. Testing peer recruitment for recruiting and effectiveness resulted in inclusion of nonrandomized elements in the peer-recruitment study. Because of privacy concerns, we were not able to track which participants recruited their peers, reducing our ability to conduct further assessment of how peer recruitment affected participants who recruited peers. Although our retention rate of 52.7% at 6 months was comparable with those of many published trials^48,49,50,51,52^ that recruited via social media and we conducted sensitivity analyses using multiple imputation, our results could be affected by nonresponse bias. We also based our 6-month smoking-cessation data on self-report. Although we collected biochemical verification for some participants, the data were not sufficient to perform subanalyses. Biochemical verification is mainly used to monitor for differential misclassification by randomization group.^40^ Studies that are in person and intense generally have more misclassification because of the personal connection between the smoker and the study team. Less misclassification occurs in low-intensity, light-touch studies in which comparisons are similar, as they were our study. Our missing data rate was similar among all groups.

## Conclusions

This randomized clinical trial may enhance understanding of novel viral recruitment methods and recommender systems for smoking cessation. Our results suggest that viral recruitment may be beneficial not only for spreading the intervention, but also for motivating smokers to quit smoking. This may open new opportunities to design digital interventions for smoking cessation as team efforts and build collaborative tools for people who smoke to not only refer, but also engage with one another throughout the intervention. Our results further suggest that through digital methods, we may have the potential to reach a larger proportion of individuals with effective methods, resulting in a greater impact. Although the recommender system in this study did not outperform the standard messaging system, the benefit of the system’s ability to continuously learn from its users may be best realized in a large and long-term implementation in which the system has sufficient duration to collect substantial amounts of feedback data.

## Notes

Supplement 1.
Trial Protocol

Supplement 2. **eTable 1.** Comparison of Baseline Characteristics Between Individuals With and Without Viral Tool Kit **eTable 2.** Comparison of Baseline Characteristics Between Individuals Randomized to ML Recommender and Standard Messaging **eTable 3.** Smoking-Cessation Rates at 6 Months for Each 3 Hypotheses With Missing Outcomes Treated as Smoking **eTable 4.** Smoking-Cessation Rates at 6 mo for 3 Hypotheses With Missing Outcomes Treated Using Multiple Imputation

Supplement 3.
Data Sharing Statement
