# Supplementary material for: Effect of a Machine Learning Recommender System and Viral Peer Marketing Intervention on Smoking Cessation: A Randomized Clinical Trial
Source: JAMA Netw Open. 2023 Jan 12;6(1):e2250665. doi: 10.1001/jamanetworkopen.2022.50665 (PMC9856644; doi:10.1001/jamanetworkopen.2022.50665)
Supplement: Supplement 1. — Trial Protocol [file jamanetwopen-e2250665-s001.pdf]

INVESTIGATOR STUDY PLAN - REQUIRED

1. TITLE
Smoker-to-Smoker (S2S) Peer Marketing and Messaging to Disseminate Tobacco Interventions

2. EXTERNAL IRB REVIEW HISTORY*
NA

3. PRIOR APPROVALS:
NA

4. OBJECTIVES*
AIM 1: Using a 2×2 factorial design with 1700 smokers, test the impact of the S2S enhanced functions to increase dissemination (recruitment and repeated use) and effectiveness of Decide2Quit.

Standard functions:
- **Online recruitment** using search engine and social media advertisements to recruit smokers to Decide2Quit.
- **Standard computer tailored health communication (CTHC)** using expert-written rules to tailor messages to smokers based on readiness to quit.

S2S functions:
- **Peer recruitment** tools to facilitate smokers' recruiting their peers to increase Decide2Quit access.
- **Recommender computer tailored health communication (recommender CTHC):** a Complex machine learning algorithm (recommender systems) that uses smokers' feedback (explicit and implicit) in the current and prior studies to adapt its selection of messages to smokers.

Study Groups: A: Fully enhanced (both peer recruitment and recommender CTHC); **B:** Recommender CTHC only; **C:** Peer recruitment only; **D:** Standard (online recruitment and standard CTHC).

AIM 2: Follow participants in the 2×2 factorial trial for six months testing S2S. We hypothesize that:

H1: Recruitment

H1A: Peer recruitment will recruit a greater proportion of African American smokers, compared to standard online recruitment.

H1B: Peer recruitment will reduce recruitment time (time to recruit each participant), compared to standard online recruitment.

H2:* Repeated use of Decide2Quit functions.

H2A: Repeated use among those exposed to the fully enhanced group (peer recruitment and recommender CTHC) will be greater than repeated use among those exposed to a) peer recruitment only b) recommender CTHC only and c) standard group (online recruitment and standard CTHC).

H2B: Repeated use among those exposed to peer recruitment will be greater than repeated use among those exposed to the standard group.

H2C: Repeated use among those exposed to recommender CTHC will be greater than repeated use among those exposed to the standard group.

H3: * Quit — six month, 7-day point prevalence— and **risk reduction** in number of cigarettes smoked (patient panel recommended outcome).

H3A: Quit rates among those exposed to the fully enhanced group (peer recruitment and recommender CTHC) will be greater than quit rates among those exposed to a) peer recruitment only b) recommender CTHC only and c) standard group (online recruitment and standard CTHC).

H3B: Quit rates among those exposed to peer recruitment will be greater than quit rates among those exposed to the standard group.

H3C: Quit rates among those exposed to recommender CTHC will be greater than quit rates among those exposed to the standard group.

H3A, H3B, and H3C will be tested for the risk reduction outcome also.

**For both H2 and H3, we will compare all smokers across the groups, and African American smokers across groups, and African American and White smokers for assessing heterogeneity of treatment effects.*

5. Background*

A.1. Overview

"**Smoker-to-Smoker (S2S) Peer Marketing and Messaging to Disseminate Tobacco Interventions**" will test two smoker-driven, social marketing innovations to recruit and engage smokers in Decide2Quit.org (Decide2Quit), an evidence-based, effective "Digital Intervention for Smoking Cessation" (DISC).[1, 2] These S2S innovations, designed to utilize the power of peers and social networks for dissemination are:

- **Peer recruitment:** Tools to facilitate smokers' recruiting their peers to increase Decide2Quit access (NCI R21CA158968).
- **Recommender computer tailored health communication (recommender CTHC):** a Complex machine learning algorithm (recommender systems) that uses smokers' feedback (explicit and implicit) in the current and prior studies to adapt its selection of messages to smokers (PCORI IP2P1000582).

Using a 2×2 factorial design, we will compare individually and collectively the enhancements (recruitment, use, and effectiveness) offered by the S2S functions over the standard version of Decide2Quit (online recruitment and standard CTHC), an active comparison group that was demonstrated to be effective in our previous trial.[3] In our prior study, we found peer recruitment was particularly effective for engaging African American smokers. We will also test the comparative effectiveness of peer recruitment to target African American smokers, a disproportionally affected group due to tobacco use, over standard online recruitment in this dissemination study.[4-10]

A.2. Condition impact (smoking) on health of individuals and populations

Smoking is the number one preventable cause of death in the United States (USA).[11-15] Yearly, over six million deaths in the world are attributable to smoking, including 480,000 in the USA.[16] The CDC estimates that for every person who dies because of smoking, at least 30 people live with a serious smoking-related illness, including cancer, heart disease, stroke, lung diseases, diabetes, and chronic obstructive pulmonary disease (COPD).[16] One estimate suggested that the proportion of USA health care expenditure attributable to smoking ranges between 6-18% of the budget across different states.[17] The cost per life year saved from use of pharmacological treatment interventions ranged between US$128 and US$1,450 and up to US$4,400 per quality-adjusted life years saved.[17] As noted in Section B.3., African Americans are disproportionately affected by tobacco and many of their health issues are directly related to their tobacco use.[4-10] Thus, reducing tobacco use will have considerable health impact, and play an important role in reducing health disparities.

A.3. Evidence gap

S2S addresses a key question — what are the effective strategies for increasing consumer demand for and use of proven, individually oriented cessation treatments, including among diverse populations? — raised in the State-of-the-Science Conference Statement on Tobacco Use.[18] We are focused on identifying effective strategies to disseminate and improve effectiveness of a DISC — www.decide2quit.org (Decide2Quit). DISCs are health communication programs accessible via Internet connections and smart phones. DISCs can include a number of functions designed to support a smoker's cessation attempt. Decide2Quit includes self-management functions, pushed and tailored motivational messages (email or text-messages), online community, and peer support. Several studies, including systematic reviews,[19-21] have shown that DISCs can be effective. A Cochrane review found a statistically significant effect comparing tailored DISCs to usual care or written self-help (RR 1.48, 95% CI 1.11, 2.78).[21] Although this evidence is mixed, two factors are typically associated with the effectiveness of DISCs.[13, 19-29] These include the use of CTHC to personalize messaging to smokers, and the engagement of smokers (recruitment and repeated system use). In our previous randomized trial [1R01CA129091] testing Decide2Quit (n=900), at 6-months, 30% of smokers who received the CTHC messages reported quitting at 6 months compared to 20% of smokers who received an interactive DISC but no messages [odds ratio 1.70 (95% CI 1.03-2.81)].[3] We have also demonstrated that the repeated use of Decide2Quit functions was significantly associated with six-month, 30-day point prevalence

cessation. Using a repeated use measure — an ordinal scale of the number of functions used after the first visit to the website (0: use of no functions, 1: use of 1-2 functions, 2: > 2-4 functions) — we found a linear association with six-month cessation. For every increase by one in this scale, odds of smoking cessation increased (OR= 2.10, 95% CI = 1.03, 4.30).[30] In conclusion, DISCs can be an effective resource for smokers, but the success depends on the implementation of CTHC, and the longitudinal engagement of smokers.

Outside healthcare, companies have innovated to use bottom-up, "user-driven" approaches to increase their websites' access and engagement. Peer marketing — engaging one customer to recruit others — has become the preferred approach to increase access to their websites. To motivate users to repeatedly use their system, companies like Amazon and Netflix Use recommender systems to deliver enhanced personalization. However, these tools have not been rigorously tested for disseminating health interventions in comparative effectiveness studies. In our prior studies [PCORI IP2P1000582, NCI R21CA158968], we have developed and demonstrated the efficacy of peer recruitment and recommender CTHC to disseminate Decide2Quit — recruit and motivate smokers to repeatedly use the system — and improve its effectiveness. Using a 2×2 factorial design, we will rigorously test these tools in a comparative effectiveness study, compared with the standard Decide2Quit (online recruitment and standard CTHC). PCORI defines dissemination as the active and targeted approach of spreading evidence-based interventions to potential adopters and the target audience through determined channels using planned strategies, and its goals as to increase the reach of information, motivation, and patients' ability to use and apply evidence.[31-33] Thus, both recruitment and use measures are needed to appropriately evaluate our DISC dissemination strategy. If recruitment is unsuccessful, then the intervention's reach is low. If recruitment is successful, but the intervention does not motivate repeated use, then there is low intervention fidelity, reducing the patient's motivation and ability to apply evidence. Beyond measuring dissemination, we will evaluate our interventions' effectiveness by measuring six-month cessation, and measuring reduction in cigarettes, as advised by our patient panel.

B. SIGNIFICANCE

Because prior literature and our own preliminary data suggest multiple barriers in the reach of smoking cessation interventions, including those online, new approaches to increasing dissemination are needed.[34] The majority of smokers are not interested in quitting at any given time.[35] Even those highly motivated to quit often fail in their attempts.[36] Peer recruitment and recommender CTHC are unique in that they harness the power of smokers to disseminate a cessation intervention (recruit and motivate to repeatedly use), and encourage cessation. In this section, we describe the potential impact and reach of DISCs, potential to target those most in need, and the two patient-centric and patient-driven S2S innovations (peer recruitment and recommender CTHC), including their theoretical foundations.

B.1. Impact of DISC (Impact = Reach × Effectiveness)

Per the RE-Aim framework,[37] an intervention's impact is a product of its reach and effectiveness. Although in-person and telephone counseling are effective, they are costly and underused (reducing their reach). DISCs can serve as important augmentation for those receiving in-person and telephone counseling (to use in between sessions and for longitudinal support). For those who do not have access to these options, DISCs may serve as the only source of tobacco cessation support. As noted, Decide2Quit achieved a cessation rate of 30% at six months, much higher than the rate (7%) at which smokers quit without support.[38] Thus, it is important to innovate and increase dissemination and effectiveness of DISCs.

B.2. DISCs: Internet, Social Media Use, and Digital divide

DISCs are health communication programs readily accessible via the Internet and smart phones. Thus, it has considerable potential to reach a large and diverse group of smokers. In 2014, an estimated 87% of Americans had Internet access.[39, 40] Digital divide in Internet access has decreased considerably, especially with increasing smart phone use. In 2015, an estimated 61% of Whites, 70% of African Americans, and 71% of Hispanics had smart phones. Most of these smart phone users had accessed a wide range of functions, including getting information about a health condition (62%) and online banking (57%). The use of online functions on smart phones is higher in lower income than higher income users.[41] Use of social media has also considerably increased.[42] Over 74% of USA Internet users use online social networks including 71% that use Facebook. Again, smart phones have decreased

the digital divide in use of social networks. A higher proportion of African Americans (48%) and Hispanics (49%) access social networks on their phones than Whites (36%).[41]

However, because of this lingering perception that vulnerable populations have little technology access, these populations are underrepresented in technology research. It may not be the lack of technology access, but that the dissemination strategies have not sufficiently evolved to engage these groups.

B.3. Increasing Reach of DISCs to African American Smokers

In a comparative effective study, we will test potential of peer recruitment to increase proportions of African American smokers in the sample. We focus on African American smokers for several reasons: 1) These smokers suffer disproportionately due to smoking-related diseases including several cancers, cardiovascular disease, and cerebrovascular disease.[4-10] Although they smoke fewer cigarettes and start smoking at an older age, these smokers are more likely to die from smoking-related diseases than Whites.[4] They are less likely to be successful at quitting than White or Hispanic smokers because they are less likely to seek cessation support, including DISCs.[4-7] 2) The standard CTHC in Decide2Quit successfully motivated African Americans smokers to quit. 3) Our NCI funded pilot demonstrated that peer recruitment significantly enriched the sample with African American smokers (11-23%). This increase happened without our tools prompting the peer recruitment of African American smokers. In S2S, with our patient advisory panel, we will further refine our instructions to encourage African American smokers' recruitment. Thus, we anticipate an even higher increase in proportion of African American smokers in S2S.

B.4. Smoker-to-Smoker (S2S) peer recruitment: Peer-referrals as an online marketing Strategy

Health information and healthy behaviors can be "infectious" — spreading between social contacts, creating cascading effects throughout the network.[43, 44] For example, over time, smokers in the Framingham cohort were less likely to smoke if someone in their network (spouse, sibling) had quit smoking.[44] Public health interventions can use these infectious effects by using engaged smokers to recruit their peers who smoke.[45, 46] Peer recruitment leverages current online marketing trends. Outside of public health, several marketing groups are using peer recruitment to enhance spread of products for a number of reasons. Customers are more likely to trust a peer referral than traditional advertisements.[47] Peer recruited customers are also more profitable than traditional customers.[47] There are several examples of successful peer-marketing (e.g., Zynga, the online social gaming company) on social media.[48, 49] To enhance the effectiveness and efficiency of these campaigns, companies have developed a more proactive approach, providing customers referral tools that allow them to easily refer others to the product (eg. email referral form).[50] The use of Facebook referral plugin in our peer recruitment system[51] was an example of this approach. Our peer recruitment experiment (NCI R21CA158968) to test whether smokers would recruit other smokers was successful. In a one-year period, peer recruitment quadrupled our sample —from 190 smokers to 759 smokers.[49, 52] Peer recruitment also increased proportions of not ready-to-quit and African American smokers (11-24%), groups that other recruitment techniques have had difficulty encouraging participation.

B.5. Computer-tailored health communication (CTHC): Standard and S2S recommender

Standard "if-then-else" theory-based CTHC systems: Theory-based CTHC is a tool that is frequently used to support behavior change. [53] It builds on the concepts of personal relevance, relatedness, and cultural similarity, constructs of multiple behavioral theories including the transtheoretical model, the theory of reasoned action, social cognitive theory, and self-determination theory.[54-56] Standard CTHC systems use selected variables from patients' baseline profile and if-then rules to send tailored messages to specific subsets of patients. [53, 57-61] These rules are developed by experts based on their knowledge of the targeted population, literature, and health behavior theories. These rules specify how the messages should be selected (what messages need to be sent to that patient subset).

<u>Recommender CTHC systems:</u> New approaches to tailoring based on collective-intelligence may be able to augment standard CTHC systems. Many people already encounter collective-intelligence tailoring as they interact with companies like Netflix and Amazon. These companies use a special class of machine learning algorithms (**recommender systems**) to tailor content. These systems tailor content based on collective-intelligence data (i.e., data derived from the behavior of users as they interact with

the system) in addition to user profiles.[62-64] Collective-intelligence data include implicit and explicit user feedback. Implicit data are derived from user actions (e.g., website pages and products purchased data). Explicit data consist of self-reported item ratings (e.g., ratings provided by users for items like books or movies, often on a five-star scale). However, in the health promotion arena, patients could be asked to rate relevance, influence, or other properties of a message or product. Using these data, along with user demographic characteristics, the algorithms underlying the recommender system generate personalized item recommendations for each user. As these recommender systems learn more about the user, they can continually adapt to improve the recommendations.

The primary difference between current and recommender CTHC systems is how the messages are selected. In standard CTHC, message selection is based on preset expert-written if-then-rules. In recommender CTHC, messages selection is based on data, i.e., recommender systems learn from the data (patient profiles and implicit and explicit feedback ratings) to select the variables and generate the rules that specify how the message will be selected. As new data about the users are collected, these recommender systems have the ability to refine the message selection algorithm. As detailed in our paper,[65] there are a number of other potential advantages of using recommender CTHC over standard CTHC systems. Our pilot experiment demonstrated that the recommender CTHC performed better than Decide2Quit's standard CTHC, a demonstrated to be effective system, even over a short duration of 30 days.

B.6. S2S Theoretical Foundations — Relatedness and personal relevance to increase engagement and motivation:

Peer recruitment: By providing peer recruitment tools to smokers, S2S facilitates smokers to reach out to their peer smokers to participate in a cessation intervention, thereby extending the intervention's reach. Peer recruitment draw strength from the constructs of relatedness, as proposed in self-determination theory (SDT).[66] Relatedness between individuals — the desire to feel connected to others — can support behavior change. [66, 67] When individuals engage in activities that are social in nature, such as providing social support, perceptions of relatedness play an important role in predicting motivation and increasing engagement.[68] Thus, both the peer recruitment act, and being peer recruited, might motivate the smoker to use the DISC, and quit smoking.

Recommender CTHC: Recommender CTHC draws strength from personal relevance, relatedness, and cultural similarity, constructs of multiple behavioral theories. Increasing the personalization of a message, increases the relevance and relatedness of the message to the user.[53] Personally relevant messages are more carefully processed, tend to be retained longer, and more predictive of behavior change.[69] By incorporating smoker's feedback, the recommender CTHC is able to adapt and continuously improve the personalization of its messages, which can increase the DISC's use, as well as cessation outcomes.

6. Inclusion and Exclusion Criteria*

All current smokers over 18 years of age, can read or speak English, and have Internet access at home will be eligible. Prisoners will be excluded. Pregnant women may be incidentally enrolled. In such cases, this research poses no risks to the fetus.

7. Study-Wide Number of Subjects*

NA

8. Study-Wide Recruitment Methods*

NA

9. Study Timelines*

Each individual subject is expected to participate in the study for duration of 6 months. The anticipated duration to enroll all study participants is with an estimated date to complete this study by December 31, 2019.

10. STUDY ENDPOINTS*

There are several data sources in this project (see Table 1 below). The primary outcome measures will be 7-day point prevalence at the 6th month follow-up (see 'S2S_6mosurvey'). Secondary outcomes include recruitment rate, patient reported outcomes collected via the 6-month follow-up questionnaire.

Table 1: Data Collection

**Data Source**	**Data Elements**	**Private identifiers**	**Data Stored In**
Search Ad Manager	Online advertisement stats (no of users who saw ads, etc.)	NO	Search ad managers (Aggregate data)
Baseline during registration (S2S_BaselineSurvey)	PCORI required demographics: age, gender, race, and ethnicity. Contact information required for follow-up: first name/nickname and telephone number.	Yes	UMass Regulated Environment
Baseline at one-week post registration (online or phone) (S2S_BaselineSurvey)	Additional demographics, Participant smoking characteristics (allowing smoking at home, quit in last 12 months), Number of cigarettes/day	No	UMass Regulated Environment
Every Decide2Quit visit	Readiness to quit, Use Of Decide2Quit	Yes	UMass Regulated Environment
Every message (email or text-messaging)(See S2S_MessageExamples)	Explicit influence ratings, message sent date	Yes	UMass Regulated Environment
1 month follow-up (online or phone) (see S2S_1MoSurvey)	Feedback on the messaging system For peer recruiters only: Peer recruitment	Yes	UMass Regulated Environment; Survey will be entered by research staff (if conducted by phone) or subjects (if completed online) directly into our server.

	experience, perceived influence of peer recruitment on cessation		
6th month Follow-up (online or phone) (see S2S_6MoSurvey)	7-day point prevalence smoking cessation, number of cigarettes	Yes	UMass Regulated Environment; Survey will be entered by research staff (if conducted by phone) or subjects (if completed online) directly into our server.
6th month biochemical verification from saliva NicAlert®	zone (0-6) indicating the level of cotinine in saliva	No	UMass Regulated Environment; Image files of the completed test will be uploaded via the Decide2Quit website, REDCap survey or emailed to a secure email address.

11. Procedures Involved*

Online Randomization: In our prior technology-assisted randomized trials, we have embedded randomization within the technology, and we will use a similar approach in S2S. As smokers register on the website, they will be allocated to the four comparators based on a block-randomization allocation table integrated into the website. Our statistician will generate a randomization table; the randomization sequence will be conducted in random blocks of different sizes (8, 12) to ensure balance among the groups and reduce predictability of allocation process. Thus, randomization will occur automatically at the time of initial registration. Peer recruited smokers will be allocated into one of the two peer recruitment arms to minimize contamination. Due to peer recruitment, we expect to recruit more African Americans in the peer recruitment group. The types of information to be asked at the baseline and 6-month surveys include demographics, smoking characteristics (level of addiction, number of cigarettes per day, smoking environment, history of quit attempts, and readiness to quit), and 7-day point prevalence smoking cessation (6-month survey only).

Comparators:

Smokers in all groups will be given access to all Decide2Quit functions. The difference in the four groups will be based on the recruitment and CTHC approaches. Our goal is to test the enhancements offered by peer recruitment and recommender CTHC over an active comparison group (recruitment using online advertisements and the standard CTHC system). We describe the comparators below.

Standard Functions

Online recruitment via search engine and social media advertisements:

Smokers will be recruited online using search engine advertisements (see S2S_Ads). These advertisements will be customized to appear to smokers searching for quit smoking related search terms online. When smokers click on these advertisements, they will be redirected to Decide2Quit, where they will be provided study information and registration instructions. We will use the functions provided in the ad managers of the search and social media websites to target ads for smokers. For example, the Facebook ad manager allows advertisers to target users based on their interests derived from their profile's keywords, pages they like, and groups they visit. Advertisements will be displayed on the Facebook page of the user. We will work with our patient advisory panel to identify relevant interests, as well as create advertisements with content that will be attractive to smokers. We will continuously monitor and refine these advertisements with our patient panel.

Other recruitment online websites include www.smoke.free.gov and www.researchmatch.org. On the www.smokefree.gov website, under the "join a research study" tab, we will provide the name of

our study, a link to the study website and a one-paragraph description of the study (see S2S_smokefree). The link listed on www.smokerfree.gov will direct smokers to the Decide2Quit website.

Research Match, www.researchmatch.org, is a website funded by the National Institutes of Health (NIH) and aims to serve as an effective and complementary tool that helps connect willing volunteers with researchers. Using research match search engine, we will search for appropriate matches (according to S2S study eligibility criteria) amongst the non-identifiable research match volunteer profiles in the system. Research match volunteers who are eligible to participant in our study will receive an invitation letter (S2S_researchmatch_invitationletter) that will provide more information about S2S. Email recipients will have the opportunity to review the invitation letter, and decide whether they would like participant in the study or not.

Standard CTHC

The difference between the standard and recommender CTHC is how messages are selected. Our study goal is to test the ability of the two systems to select influential messages for individual subjects. Thus, both messaging systems will use the same message database. We plan to send 2 messages weekly in the first four weeks of the intervention. We will then send 1 message per week until the smoker completes six months in the intervention (from registration date of a smoker). Below, we first describe the messaging database and then the standard CTHC system.

The Motivational Messaging database:

The messaging database includes 500 messages that were developed in our prior RCT and includes both expert-written messages and peer-written messages.[73] Expert-written messages (behaviorists, physicians, nurses) were developed through an iterative expert group review process. These messages were informed by current guidelines[74] and Social Cognitive Theory.[75] The current guidelines provided evidence-based content on successful cessation strategies. The Social Cognitive Theory, which incorporates vicarious learning and verbal persuasion, guided the writing of the expert messages. Messages reflect theoretical determinants of quitting, such as positive outcome expectations and self-efficacy enhancing small goals.[75] Peer-written messages were written by current and former smokers responding to an online survey that presented four scenarios tailored by gender, age and readiness-to-quit. These messages were then reviewed for use in our system. More details of our methodology to generate peer written messages has been previously published.[73] (See S2S_MessageExamples).

Standard Decide2Quit CTHC System:

As noted, our comparison standard CTHC is a rule-based (if-then-else) system that tailors messages based on a smoker's readiness to quit. For example, when a smoker logs on to Decide2Quit and indicates his readiness as "not ready to quit", then a message from those categorized for "not ready to quit" smokers will be picked at random and sent to the smoker. Similarly, if the smoker indicates his readiness as "set a quit date", then a message categorized for "set a quit date" smokers will be sent to the smoker. The messages can either be sent via emails or text messages.

S2S Functions

Peer Recruitment:

Peer recruitment will start with the standard online recruitment of seeds (first wave). These seeds and subsequent waves of smokers (peer recruits) will be provided the peer recruitment toolset to recruit others. This toolset will not be used until we have IRB review and approval. The peer recruitment toolset includes a Facebook website plugin, an online training video, and a recruitment tracker. Our Facebook website plugin was developed in our NCI-funded pilot (R21CA15896).[49, 51] The Facebook plugin will allow smokers to browse through their Facebook friends and recruit them by sending private recruitment messages. Smokers will also be able to peer recruit through text messages and emails. The online training video will teach the recruiter how to use available tools to recruit other smokers from their social network. The recruitment tracker will allow smokers to track successful peer recruitment. To encourage recruitment, the system also will email or text a weekly recruitment report to the potential recruiter. Peer recruitment will happen in waves. As is common in peer recruitment approaches, to initiate the waves, we will recruit the first wave (wave 0 or seeds) of smokers (both current and former smokers). Similar to comparison, seeds will be recruited using online

advertisements. Once a seed registers on Decide2Quit and receives the peer recruitment toolset, we expect the following steps:

1. The seed consents to be in the study (See S2S_Consent) and recruit smokers from his/her network using the peer-recruitment tools (by sending a Facebook private message).
2. The successfully peer-recruited smoker (Wave 1 recruit) registers on the system and consents to then recruit other smokers in their social network.
3. The Wave 1 recruit then continues the peer-recruitment chain, recruiting smokers from their social network.
4. The successfully peer-recruited smoker then registers (Wave 2 recruit).
5. The waves progress until the target sample size is reached.

In addition to supporting the smokers' ability to recruit via Facebook by sending a message, we will also support recruitment messaging via emails and text-messaging to further expand reach in this study.

Recommender CTHC system:

The difference between the standard and the recommender CTHC is the approach to message selection and not the messages. We will use the same motivational messages as the standard CTHC system. Our recommender CTHC system is a hybrid recommender system. We chose a hybrid approach because they merge the strengths of content-based and collaborative filtering recommender systems.[76] Thus, they can potentially benefit from expert-driven rules (content-based), and the recommender algorithms. Our hybrid recommender system uses three input data sources to generate the recommendations, including the 1) metadata description of the messages, 2) implicit, and 3) explicit feedback data (smokers in prior and current study). Our metadata includes a comprehensive coding of the messages. Implicit feedback data are derived from user actions. As our implicit feedback data, we used the website return data of 900 smokers that participated in our prior RCT.[3] When an email was sent to these smokers, we tracked their website usage in the days following the email. Thus, we had data on the frequency at which each message promoted use of Decide2Quit, and the characteristics of the smokers that received these messages.

Explicit feedback data consists of self-reported item ratings. Two pilot studies were used to generate the explicit feedback data for recommender CTHC.[77] In addition to explicit feedback ratings from smokers in prior studies, the recommender CTHC is programmed to also use the explicit ratings of smokers receiving the messages. Thus, when a smoker is sent an email, we will include a link to rate the message on the influence scale. Although our standard CTHC does not adapt to this feedback, we will include the ratings question in the standard CTHC messages to minimize group differences.

Patient-centered outcome measures:

S2S will include multiple data collection stages (Table 2). At each time point (one week, one-month and six months) we will send participants email reminders to complete the online follow-up surveys. We will send up to 4 email reminders over the course of two weeks from the targeted follow-up date (e.g., if the participant is due for their 6 month follow-up on January 1st, we will send them reminders for the two weeks following that date). If participants fail to respond to our email messages, we will call them to complete the survey over the phone. For all participants (N=480) who did not initially respond to our six-month follow-up correspondence, we would still be seeking to ascertain the original end-point of six-month follow up and would be extending the follow-up window to potentially a maximum of 18 months. We will target participants who are more than 2-3 months outside their six-month follow-up window.

We are planning to start calling them 2 months after their six-month window closes. We will send 1 email followed by 4 phone calls over a course of three weeks. At the end of the 4th call attempt we are planning to leave a short voice mail, including our name and state that we will call again. (We will leave our phone number if the participant would prefer to call us back). These phone calls will be completed between business hours (10:00am-4:00pm), excluding early mornings. To see the text for the email invitations and the intro script for the telephone calls refer to the S2S_Invitation_For_Followup.docx.

All participants (N=704) who completed the six-month follow-up survey will be offered the opportunity to voluntarily participate in an online survey. *(COVID-19 related smoking survey_5.4.2020).* Primary purpose of this survey is to understand how smokers (and vapers) are perceiving the risks of COVID and to evaluate how social isolation has impacted their lives and mental health and how all this has affected their smoking and vaping.

Survey will be sent to participants via email, 4 times, over a two-week period *(S2S email Invitation_COVID19_4.24.2020)*. Compensation will be provided in a form of $10 gift card upon completion of the survey.

We developed our outcome measures based on over 15 years of DISC research with smokers.[2, 51, 52, 78-80] To appropriately measure dissemination, we need both recruitment and use measures. We also will test effectiveness in this study. Our primary measures are described below:

Measures:

Recruitment time: When smokers register on Decide2Quit, they will be assigned a unique identifier and their registration date and time will be recorded. We will compute recruitment time from this data as the time taken to recruit each participant from the time that the first participant in the group was recruited. (see equation below).

$$\textbf{Recruitment time } T_i = \mathrm{X_i} - \mathrm{X_1}$$

where $\mathrm{X_i}$ is date of registration of $\mathrm{i^{th}}$ participant; $\mathrm{X_1}$ is the date of registration of $\mathrm{1^{th}}$ participant.

Repeated Use: We use repeated use over other use measures (number of logins) because of the demonstrated association with smoking cessation.[30] This is an ordinal scale of number of Decide2Quit functions used after the first DISC visit (0: no functions used; 1: use of 1-2 functions, 2: > 2 functions used).

Effectiveness Measures:

7 day point prevalence cessation: 7 day point prevalence will be assessed at six months using: Do you currently smoke cigarettes (smoked even 1 puff in the last 7 days)?[82] The 7-day window provides an appropriate stringent measure to account for a cross-sectional snapshot.

Table 2: Key Data Elements	Search Ad Manager	Baseline during registration or one week	Every Decide2Quit visit	Every email message	6th month Follow-up (Online or phone)
Online advertisement stats	X				
Demographics		X			
Participant smoking characteristics		X			
Number of cigarettes/day		X			X
Readiness to quit		X	X		
Use of website			X		
Explicit influence ratings				X	
7-day point prevalence					X
Biochemical verification saliva NicAlert®					X

Optional biochemical verification by saliva test NicAlert®: Subjects indicating that they have quit at the 6-month follow-up survey will be given the chance to provide biochemical verification using the NicAlert® test (Nymox Corporation). NicAlert® is a semi-quantitative method that uses a dipstick to measure the level of cotinine in a sample of saliva. The test strip displays the result in seven zones. Each zone represents a range of levels of cotinine/smoking [e.g. zone 0 (0–10 ng/mL, a nonsmoker) to zone 6 (>1000 ng/mL, a heavy smoker)]. The results will be read as 0-6, and as recommended, any value ≥ 1 will be considered as tobacco use.[83-85]We will mail the strips with clear instructions on how to take a picture and return the picture of the results to us electronically. Our staff will also be available by phone to help the smokers complete testing.

Risk reduction or the reduction in the number of cigarettes smoked: We will calculate risk reduction using the below formula:

Risk reduction = Number of cigarettes smoked at follow-up – Number of cigarettes smoked at baseline

12. Data and Specimen Banking*

NA

13. Data Analysis and Management*

Sample Size and Power Calculations:

H1A:

In our NCI-funded pilot (R21CA15896),[49, 51], peer recruitment increased the proportion of African Americans to 23%, compared to 11% in the initial seeds (those recruited by advertisements). Using 10% as the base rate in the non-peer recruitment group (recruitment by online advertisements), we estimated sample size requirements varying the proportion in the peer recruitment from 16 to 20%. With these assumptions, we will need 219 smokers in each group to detect difference of 10% (power = 80%, alpha=.05). If we reduce the difference to 8% and 6%, we will need 319 and 525 in each group respectively. Given that we will work with our panel to encourage recruitment of African American smokers in the peer recruitment group and may see bigger differences than our pilot, we will have adequate power particularly with the proposed sample size of 600 in each recruitment method.

H1B:

In the NCI pilot (R21CA15896),[49, 51] we estimated that the mean number of days to recruit a sample of 700 smokers was 244 days with a standard deviation of 81. Assuming that peer recruitment proceeds with the same rate and with the same standard deviation, we can detect a difference in recruitment time as low as 14 days. Since we expect the comparison rate to be much slower, we are adequately powered to detect differences with a sample of 600 (power=0.8). Calculations were made in STATA.[89]

H2:

We used the method published by Whitehead to calculate power for this hypothesis.[90] As noted above, in our prior RCT (1R01CA129091), we found a linear association with six-month cessation using the repeated use scale. For every increase by one in this scale, odds of smoking cessation increased (OR= 2.10, 95% CI = 1.03, 4.30),[30] with the current sample size of 300 per group for H2A, we can detect a difference a cumulative odds ratio of 1.7. Thus, our study is adequately powered to measure a reasonable difference in the repeated use measure. Power for these hypotheses is driven by H2A, as this analysis is looking at the smaller sample (fully enhanced). For H2B and H2C, power is even greater, as the sample size is 850 per group for each.

H3:

Quit: We assumed a control cessation rate of 15%,[91] a two-sided significance level of 0.05. A sample size of 300 in each group (H3c) will achieve 80% power to detect a difference of 9% (quit rate in intervention=24%) in quit rates between the two groups, based on a Z-test with pooled variance. We will categorize the NicAlert® test results into smokers and non-smokers and use the Chi-Square statistic to test for differences.

Risk Reduction*:* We calculated the detectable difference in risk reduction with 300 smokers in each group and the mean in the comparison group of 3.3 using standard deviations of 2 and 3 with 80% and 90% power. We will have 90% power to detect a difference of 0.80 (or smaller) number of cigarette smoked reduction between two groups. This difference is likely to be achieved based on the results of our PCORI pilot in which we achieved a reduction of 0.85 (4.15 to 3.3) in 30 days; compared to smokers receiving the standard CTHC messages, smokers receiving the recommender CTHC had a higher reduction in number of cigarettes at 30 days (Standard CTHC: mean 3.3; *S2S adaptive CTHC*: 4.15).

Analytical Plan

To preserve randomization, all primary analyses will be on an intent-to-treat basis. Secondary analyses will explore dose-response effects among those with variable levels of adherence to the intervention. All analyses will be two-sided and alpha error will be set at 0.05. We will begin our analysis

by examining univariate statistics (means, medians, standard deviations and 95% confidence intervals) and distributions. We will examine the balance of participant characteristics by study groups and account for any imbalances in our multivariable analysis. The 2x2 factorial design will result in 4 study groups (**A: Fully enhanced; B: Recommender CTHC only; C: Peer recruitment only; D: Standard**). As appropriate, differences in measured characteristics (i.e., demographics and pre-baseline smoking behaviors) by group will be tested using chi-square tests of independence (categorical variables), ANOVA (continuous variables) or the equivalent non-parametric tests depending on the distribution of the variables. Per best practice, differences in baseline characteristics of the intervention and comparison groups will be assessed.[92, 93]

<u>H1A:</u>

Based on our pilot, we anticipate that peer recruitment will be more effective in recruiting African Americans. To test this, we will categorize the smokers as either African Americans or not, and then use the Chi-Square statistic to test for differences between the peer recruitment and standard groups

<u>H1B:</u>

We will compare mean recruitment time between the two types of recruitment method using a t-test. We will then develop a linear regression model to adjust for possible confounders such as covariates that are not balanced between the smokers recruited from the two methods as well as important predictors of recruitment. <u>Within the peer recruitment group,</u> we will conduct a secondary analysis examining differences in demographic characteristic between peer recruited and directly recruited smokers. Using data provided by search engine advertisement managers, we will evaluate the performance of our online advertisements (number of users registered on Decide2Quit following an advertisement on the search engine).

<u>H2:</u>

As noted above, we will have four study groups: (A: Fully enhanced; B: Recommender CTHC only; C: Peer recruitment only; D: Standard). We will use the following generalized linear model, which includes indicators of peer recruitment and recommender CTHC and the interaction between the two indicators as independent variables, to test H2A:

$$E(\boldsymbol{f}(\boldsymbol{u})) = b_0 + b_1 \times P + b_2 \times R + b3 \times P \times R + potential\ confounders\ (demographics, readiness\ to\ quit)$$

where u is the outcome measure (repeated use measure), the function $f(u)$depends on the distribution of u,

P=1 for peer recruitment, =0 for standard recruitment, *R*=1 for recommender CTHC, =0 for standard CTHC.

The adjusted $f(u)$ (i.e., adjusting for the potential confounders) for each of the 4 groups can be expressed in terms of the regression coefficients defined in the box below.

A	B	C	D
$b_0 + b_1 + b_2 + b_3$	$b_0 + b_2$	$b_0 + b_1$	b_0

In the model, $b_2 + b_3$ is the estimated difference between group A (peer recruitment and recommender CTHC) and group C (peer recruitment only); $b_1 + b_3$ is the estimated difference between group A and group B (recommender CTHC only); and $b_1 + b_2 + b_3$ is the estimated difference between group A and group D (standard). Significant positive values of the estimated differences will support H2A. In the event that there is an interaction effect between the two S2S enhancements (peer recruitment and recommender CTHC), i.e., b3 is significantly different from zero, H2B and H2C will be tested by comparing groups C vs. D (peer recruitment alone vs. standard Decide2Quit; estimated by b1) and groups B vs. D (recommender alone vs. standard Decide2Quit; estimated by b2). If there is no interaction effect, i.e. b3 is not significantly different from zero the following model will be used to test H2B and H2C:

$$\boldsymbol{E}(\boldsymbol{f}(\boldsymbol{u})) = a_0 + a_1 \times P + a_2 \times R + potential\ confounders\ (demographics, readiness\ to\ quit).$$

In the model, a_1 is the estimated effect of peer recruitment [(A+C) – (B + D)]; a_2 is the estimated effect of recommender CTHC [(A+B) – (C+D)]. A significant positive value of a_1 or $a_2$will support the hypotheses of positive effects of these two individual components respectively (H2B and H2C).

We will examine the distribution of the dependent variable u to determine the link function to be used in the generalized linear model. In our previous study, we used an ordinal variable for the dependent variable Repeated Use with log link function to fit an ordinal logistic regression.[30]

We will also conduct a secondary analysis using the influence ratings. As noted above, both standard and recommender CTHC will include a link to rate the messages on the influence scale. For this analysis, the dependent variable for each smoker is the mean of all influence ratings (an influence score) and the independent variable is study arm [recommender (A+B) or standard CTHC (C+D)]. We predict that the mean influence score will be higher in recommender CTHC than the standard CTHC arm. For this analysis, we will start with a t-test for differences in mean, but acknowledge that the influence scores may not be normally distributed and only approximate a continuous variable. Thus, we will use a Wilcoxon rank-sum.

As noted, for both H2 and H3, we will also compare African American smokers across the groups, and African American and White smokers to test for heterogeneity of effect. This will provide us important data for design future interventions.

14. Provisions to Monitor the Data to Ensure the Safety of Subjects*

Data and Safety Monitoring Plan

The risks associated with the data collection and participation in this study are not high. To that end the purpose of this Data and Safety Monitoring Plan (DSMP) is to specify the procedures and rationales of the current study to ensure the safety of participants' data and the validity and integrity of the data.

The primary risk to subjects is the accidental disclosure of information; however, every precaution will be taken to prevent this and the study team has an excellent track record of protection of confidential data. The data will be stored in a HIPAA compliant UMass regulated environment and access will be only through a secure VPN network. The UMASS Medical School Regulated environment provides applications a secure network for confidential data. The Regulated environment has been securely configured to allow application access via the secure socket layer (HTTPS) protocol — a protocol that delivers server authentication, data encryption, and message integrity. The setup of Regulated environment provides the needed security protocols for the regulatory and Federal standards required. The Regulated environment is secured using hardware and software firewalls, along with access restrictions to enforce governmental policies requiring to enforcement.

In no way will individual participant data be released to the public or cited in a publication. Our group has substantial experience with implementing these methods successfully. Our interaction with participants is minimal, and all data will remain confidential and only reported in aggregate.

To further mitigate risk have minimized data collection to that which is needed to answer the study hypotheses. We will have stringent protection against breach of confidentiality using secured servers and locked office spaces for data entry at UMMS. The research coordinator will do periodic checks to ensure that participant confidentiality is protected at all stages of data management and analysis. At the start of the study all project team members will be trained in practices that ensure participants' confidentiality and privacy. There are no health or safety risks for participation in the S2S study.

To monitor the validity and integrity of the data, the Decide2Quit platform has a database administration site. The admin site will contain data collection reports to be used for the ongoing monitoring of data in the database. The PI, Dr. Sadasivam, the biostatistician Dr. Chang, and the analyst Ms. Orvek will regularly review database admin reports to inspect for data collection completeness. Furthermore study recruitment and retention will be monitored using the aggregated summary reports available on the admin site.

15. Withdrawal of Subjects Without Their Consent*

NA

16. Risks to Subjects*

The risks of the study are not high, and thus the safety monitoring plan has been matched to the risk to subjects in this study. The major risk is the accidental disclosure of information; however, every precaution will be taken to prevent this and the study team has an excellent track record of protection of confidential data. There may be some stress involved in completing the follow-up survey, but we will emphasize that this participation is voluntary.

17. Potential Direct Benefits to Subjects*

Potential Benefits of the Proposed Research to the Subjects and Others

The major benefit to smokers is the additional resources to encourage smoking cessation and the potential for supporting cessation attempts resulting in quitting smoking and the resulting health benefits.

18. Vulnerable Populations*

NA

19. Multi-Site Research*

NA

20. Community-Based Participatory Research*

NA

21. Sharing of Research Results with Subjects*In addition to the dissemination methods described below, we plan on sharing results with study subjects as instructed by PCORI. We will do so by sending a summary of aggregate findings containing no identifiable information once the study is completed to the email address provided by subjects at the time of enrollment.

Additional dissemination methods of research results include:

- Presentation and Publications: We have an exemplary track record in publishing and presenting results from our funded studies. We will diligently apply this experience and skill to disseminate S2S results. In addition to presenting our findings at scientific meetings and in peer-reviewed literature, we will also present key findings at community-based meetings, on our webpage, and social media sites. Our patient panel will be an integral part of this effort.

- Community-based outreach: With our patient panel, we will develop lay summaries and marketing materials and start identifying opportunities for continued dissemination of Decide2Quit and S2S tools in community-based settings. Dr. Allison (key investigator) is the lead of the Special Population Resource Center, of the UMass Clinical and Translational Science Center, as well as the Vice Provost for Health Disparities research. Under his leadership, UMass is developing relationships with several local and national community organizations (MOSAIC is one example). Dr. Allison will continue to support our efforts to identify different groups with whom we can collaborate to disseminate Decide2Quit. We have specifically recruited the MOSAIC cultural complex to serve in our panel to be able to start building bridges with local communities and explore opportunities to disseminate Decide2Quit. MOSAIC is working with several ongoing studies at UMass and is committed to the effort of integrating evidence-based tools into their communities to help decrease health disparities.
- Integration with EHR/PHR and other healthcare settings: We will also take a proactive approach to dissemination of the tools leveraging our past research. In our prior RCT, [2, 3, 94, 95] we tested an electronic referral system to market Decide2Quit. As smokers were seen by their primary care providers, providers were trained to refer them into Decide2Quit website by entering their email address into a secure form. Once e-referred, Decide2Quit website sent them up to 10 emails encouraging their participation into the study. This project was highly successful. We had 2166 referrals from 74 practices, out of which, 672 smokers registered. The next step in this approach is to link the e-referral to EHRs around the country. We have several national connections with the Veterans Health Administration (VHA) and healthcare systems through our Clinical and Translational Sciences Award. Through these connections, we will explore adapting the e-referral system to their environment. In our PCORI pilot (1IP2P1000582), we also experimented with identifying smokers in the EHR and mailing them for participation in the study. As the majority of smokers see a healthcare provider, this is potentially a powerful approach for disseminating Decide2Quit tools. We will again make use of our national connections to assess interest in this approach in various healthcare systems.
- Other social media opportunities: During initial conversations, our patient panel members were excited about the use of social media outlets (e.g., Twitter, Facebook) for dissemination and outreach with study participants (see below). Dr. Sadasivam is the social media chair of the Society of Behavioral Medicine and has expertise in developing social media campaigns. For example, we will create a twitter group and work with our patient panel to develop tweets. In previous work,[96] we found that Twitter tobacco cessation groups whose tweets had a higher frequency of socioemotional support and encouraging/engaging tweets had higher followership. We will use these findings to increase the followership of our group. Our study participants will also be invited to subscribe to social media channels after they complete the study (see below), and we will use these mediums to disseminate study results. We will also feature the intervention as part of the social media outreach program of our National Institute on Minority Heath and Health Disparities funded Center for Health Equity Intervention (CHEIR). Dr. Allison is the lead of CHEIR, and Drs. Sadasivam and Houston play a lead role in shaping the social media outreach of CHEIR.
- Other media opportunities: Dr. Houston's previous interventions have been incorporated into the Innovations Exchange. Our work have been featured in the in the New York Times,[97] the Boston Globe,[98] Time Magazine,[99] and Wall Street Journal.[100] We will continue to explore opportunities to have our results featured in these outlets.
- Technological advancements to support dissemination: As noted (Section C.6.3), we will pursue technological advancements of the S2S tools to further their dissemination potential. With our colleagues at the Center for Intelligent Information Retrieval (CIIR) at UMass Amherst (Dr. Marlin), we will develop targeted peer recruitment strategies using algorithms to identify potential "best" recruiters. Instead of pursuing everyone, this will allow dedicating resources to further the potential

of these recruiters. This will also support targeting those that can best reach a vulnerable population. We will continually work to enhance the recommender CTHC algorithm, including tailoring the timing of the message delivery, and incorporating additional real-time information from sensors.

22. Setting

Smokers will be recruited online using search engine advertisements. These advertisements will be customized to appear to smokers searching for quit smoking related search terms online. When smokers click on these advertisements, they will be redirected to Decide2Quit, where they will be provided study information and registration instructions. We will use the functions provided in the ad managers of the search and social media websites to target ads for smokers. For example, the Facebook ad manager allows advertisers to target users based on their interests derived from their profile's keywords, pages they like, and groups they visit. Advertisements will be displayed on the Facebook page of the user. We will work with our patient advisory panel to identify relevant interests, as well as create advertisements with content that will be attractive to smokers. We will continuously monitor and refine these advertisements with our patient panel. All additional study activities will occur virtually, such as via phone calls, emails, and text messaging. Data analysis will be conducted in a private setting at UMass Medical School. Analyses will be completed using UMass protected computers in a secure environment.

23. Resources Available

Principal Investigator: (will devote 25% effort or 3.0 calendar months in years 1 and 2 and 25% effort or 2.25 calendar months for 9 months in year 3 of the project due to the dedicated effort for peer review) The PI is an Assistant Professor in the Division of Health Informatics, QHS. The components in this grant were developed in the PI's prior grants. The PI was the PI of the Share2Quit chain referral study (NCI R21CA158968), in which he developed and evaluated the S2S peer recruitment tools. Based on this work, he published 2 manuscripts, and a 3rd manuscript is under review. He is also a recent awardee of a National Cancer Institute K07 award (2nd year). The goal of his NCI K07 award is to train in cancer health behavior, health communication, and technology intervention design, and analysis to further innovate on the S2S adaptive CTHC system. As such, the SES proposal is very much in line with the PI's training and research goals. The PI will be responsible for the overall conduct of the study.

Co-Investigator #1
(Co-Investigator #1 will devote 20% effort or 2.4 calendar months in all years of the project) Co-Investigator #1 has gained national recognition for his health informatics research with a specific focus on patient informatics and is current director of the Behavioral Informatics special interest group of the Society for Behavioral Medicine. Co-Investigator #1 has been PI of two NIH-funded R01 tobacco control grants (R01DA017971 and R01CA129091). He has published articles on tobacco epidemiology, social networking, health services delivery, research methods, and intervention to reduce tobacco use. Because of his specific expertise in tobacco control and technology assessment, Co-Investigator #1 was asked to be a Deputy Editor of the prestigious journal, *Medical Care*. He is a fellow of the Society of Behavioral Medicine and the American Medical Informatics Association. Co-Investigator #1 was key personnel in our NCI funded Share2Quit chain referral study (NCI R21CA158968), which this project builds on. He was also the PI of the PCORI project in which we developed the S2S adaptive CTHC system (IP2P1000582). Co-Investigator #1 and the PI have collaborated for over 12 years developing the Decide2Quit.org DISC and the S2S functions, and will continue their collaborations in this grant. This individual will neither interact with subjects nor access private identifiable information about them.

Co-Investigator #2: (will devote 10% or 1.2 calendar months in all years of the project)

Co-Investigator #2 is the director of the UMass Center for Health Equity Intervention Research (CHEIR), which is funded by the National Institute of Minority Health and Health Disparities to both develop new disparity-reducing interventions and to enhance the diversity of the biomedical research force by training the next generation of scientists from under-represented backgrounds. Co-Investigator #2 has made an increasing commitment to health disparities research and health equity interventions and has a strong interest in understanding the root causes of health disparities and developing community-informed interventions based on this understanding. In this project, Co-Investigator #2will advise the PI on how best to target recruitment and engagement of African American study population. This individual will neither interact with subjects nor access private identifiable information about them.

Statistician: (will devote 25% effort or 3 calendar months in year 1, 10% effort or 1.2 calendar months in year 2, and 25 % or 3.0 calendar months in year 3 of the project)
The Statistician has a broad, yet robust background in statistics and survey methods. She has experience applying statistical techniques to the fields of epidemiology and health services research, and to topics within each of these, including measurement/surveillance of disease outcomes, disparities in health care, adoption of Electronic Health Records, impact of clinical tools, and survey research, and global health. A senior faculty member of the Quantitative Methods Core, the Statistician will direct the statistical analyses for evaluating processes and outcomes. This individual will neither interact with subjects nor access private identifiable information about them.

Consultant: (will devote 15% effort or 1.8 calendar months in all year 1 and 3, and 1.2 calendar months or 10% effort in year 2 of the project)
The Consultant is the director of the Tobacco Consultation Services at the UMass Memorial Medical Center (UMMC). He is also an Adjunct faculty at the Department of Psychiatry at the UMass Medical School. He has been certified as a Master Level Tobacco Treatment Specialist for over 11 years and holds additional certifications and licenses as an Addictions Specialist, an Independent Alcohol and Drug Counselor and Social Worker. In his role as a tobacco treatment specialist at the UMass Memorial Medical Center, he counsels smokers daily at both inpatient and outpatient settings. The consultant has extensive experience in tobacco dependence treatment in his role in oversight of the tobacco and smoke-free campus movement for both UMass Memorial Health Care and the University of Massachusetts Medical School. The consultant will participate in our patient and stakeholder advisory panel, and will provide input on all phases of the project. This individual will neither interact with subjects nor access private identifiable information about them.

Programmer: (will devote 30% effort or 3.6 calendar months in year 1, and 15% effort or 1.8 calendar months in years 2 and 3 of the project)
The programmer has developed the S2S tools under the supervision of the PI. In this project, her role will be limited to integrating the new randomization design in the D2Q DISC (year 1), and for maintenance of the system in years 2 and 3.

Analyst:
Under the supervision of Biostatistician, the analyst will conduct the statistical analysis for the three hypotheses described in the proposal at 25% effort or 3 calendar months in year 1, 10% effort or 1.2 calendar months in year 2 and 50% or 6 calendar months in year 3.

Project Director: (will devote 50% effort or 6.0 calendar months in all years 1-2, and then 50% effort or 4.5 calendar months in year 3 for 9 months due to the dedicated effort during the peer review period of the project)

The project director will be managing our Research Coordinator and Assistant to implement all aspects of the study protocol including the follow-up, data collection, overseeing the distribution of incentives to participants, and directing the study staff in any other needed areas. Along with the PI, the project director will work with the patient and stakeholder researchers to ensure that they understand all aspects of the research study.

Research Coordinator #1: (will devote 100% effort or 12.0 calendar months in year 1-2, and 50% or 4.5 calendar months for 9 months due to the peer review period in year 3 of the project.
Under the direction of the project director, Research Coordinator #1 will participate in all phases of patient recruitment and patient telephone follow-up. She will be an active study staff member and will specifically be responsible for coordinating participant follow-up. She will work closely with PI and project manager to design and print materials required for the project and prepare all IRB and human use protocol materials, ensuring annual renewal of IRB approval.

Research Coordinator #2: (will devote 50% or 6.0 calendar months in year 1 and 9 calendar months or 75% in years 2 & 3 due to the data collection in year 2&3)
Research Coordinator #2 is an active study staff member who will assist in patient recruitment, patient follow-up and data entry. She will work closely with project personnel to provide needed administrative support for all study tasks.

Patient Investigators: (will attend 15 1-hr meetings per year in all years of the study)
In order to maximize the effectiveness of the intervention, we will have 5 patient investigators join the research team. All patient investigators/stakeholders will participate in an advisory panel and participate in all aspects of the study, including 1) refining our online recruitment strange (in terms of content or where to place the ads); 2) Monitoring our implementation progress and helping us refine our approach in case of issues such as slow recruitment; 3) Evaluating the data and helping us present results to smokers and community-based organization; and 4) helping us prepare for approaches to disseminate the intervention beyond the scope of the project. These individuals will neither interact with subjects nor access private identifiable information about them.

Under the close supervision of the Principal Investigator and the Project Director, all research study staff will be appropriately trained and will review the protocol to inform them of their duties and ensure proper conduction of the research study.

24. Local Recruitment Methods

We will recruit 1700 smokers (425-440 smokers per group) for this study. Smokers will be recruited through online advertisements (see S2S_Ads) (Facebook and Google) or by peer recruitment. These smokers will be asked to provide online consent (See S2S_Consent). We have conducted multiple online studies and will format our online consent to be easily understandable to the participants. (see #22 Setting for more information about online recruitment method)

25. Local Number of Subjects

Recruitment of all subjects will occur online and will not be restricted to local recruitment.

26. Confidentiality

We have minimized data collection to that which is needed to answer our hypotheses. We have stringent protection against breach of confidentiality using secured servers and locked office spaces for

data entry at UMMS. The study PI and research coordinator will do periodic checks to ensure that participant confidentiality is protected at all stages of data management and analysis. All data collected will be stored in a HIPAA compliant regulated environment and access will be only through a secure VPN network. All smokers' related identifiers are encrypted in the database. Our biostatistician will organize data security and archiving. In no way will individual participant data be released to the public or cited in a publication. We have substantial experience with implementing these methods successfully. All our research staffs are trained in HIPAA compliance and will complete all human subjects training. Our grant funder, PCORI, may also be an entity that can access confidential information within our study.

To ensure that the study data is held confidential, consented participants will be assigned a study ID (identifier). We will not be collecting Social Security numbers, Date of Birth, or other unique identifiers other than those previously mentioned. All study data will be entered and stored on a secure server that that is assigned to the Department of Quantitative Health Sciences. Only UMMS IRB and study investigators will have access to the research data. The IP address of the subjects' computers will also be retained and kept in a secure location. IP addresses will be linked only to study participant code numbers. This information, along with all other data on enrolled participants will be destroyed 5 years after the protocol ends.

27. Provisions to Protect the Privacy Interests of Subjects

At the start of the study, all study staff will be trained in practices that ensure participants' confidentiality and privacy. There may be two situations that could accidently reveal the participation in an online study. Our Facebook website plugin will facilitate peer recruitment of smokers to the study. Through the website plugin, smokers will be able to post private messages. These private messages are only viewable by the person that the message is sent to. We will emphasize with the recruiter the importance of using private messages, and the potential risks of posting messages on Facebook through other means. No identifiers of the person being peer recruited will be collected until the person enrolls in the study.

28. Compensation for Research-Related Injury

This research does not involve more than minimal risks to participants.

29. Economic Burden to Subjects

There is no economic burden to participants.

30. Consent Process

Individuals who are interested will be directed to a website that will explain the study in detail. Once the individual has agreed to participate they will be presented a webpage with a consent form. If they click on "yes" they will agree to participate in the study. Contact information for both the PI and the research coordinator will be available on the consent form.

31. Process to Document Consent in Writing

We have requested a waiver of written consent due the minimal risk of the study and involves no procedures that require written consent outside of the research context.

32. Drugs or Devices

NA

REFERENCES

1. *Houston, T.K. and Ford, D.E., A tailored Internet-delivered intervention for smoking cessation designed to encourage social support and treatment seeking: usability testing and user tracing. Inform Health Soc Care, 2008. 33(1): p. 5-19. PMID:*

2. *Houston, T.K., Sadasivam, R.S., Ford, D.E., Richman, J., Ray, M.N., and Allison, J.J., The QUIT-PRIMO provider-patient Internet-delivered smoking cessation referral intervention: a cluster-randomized comparative effectiveness trial: study protocol. Implement Sci, 2010. 5: p. 87. PMID:*

3. *Houston, T.K., Sadasivam, R.S., Allison, J.J., Ash, A.S., Ray, M.N., English, T.M., Hogan, T.P., and Ford, D.E., Evaluating the QUIT-PRIMO clinical practice ePortal to increase smoker engagement with online cessation interventions: a national hybrid type 2 implementation study. Implement Sci, 2015. 10: p. 154. PMID:*

4. *Centers for Disease Control and Prevention. African Americans and Tobacco Use. 2014; Available from: http://www.cdc.gov/tobacco/disparities/african-americans/index.htm.*

5. *Kulak, J.A., Cornelius, M.E., Fong, G.T., and Giovino, G.A., Differences in Quit Attempts and Cigarette Smoking Abstinence Between Whites and African Americans in the United States: Literature Review and Results From the International Tobacco Control US Survey. Nicotine Tob Res, 2016. 18 Suppl 1: p. S79-87. PMID:*

6. *Nollen, N.L., Cox, L.S., Yu, Q., Ellerbeck, E.F., Scheuermann, T.S., Benowitz, N.L., Tyndale, R.F., Mayo, M.S., and Ahluwalia, J.S., A clinical trial to examine disparities in quitting between African-American and White adult smokers: Design, accrual, and baseline characteristics. Contemp Clin Trials, 2016. 47: p. 12-21. PMID:*

7. *Webb, M.S., Francis, J., Hines, B.C., and Quarles, F.B., Health disparities and culturally specific treatment: perspectives and expectancies of African American smokers. J Clin Psychol, 2007. 63(6): p. 567-83. PMID:*

8. *Webb, M.S., Science Brief: Addressing Tobacco-Associated Health Disparities through Behavioral Science, in Psychological Science Agenda, A.P. Association, Editor. 2010*

9. *American Cancer Society. Cancer Facts & Figures for African Americans. [cited 2016 Apr]; Available from: http://www.cancer.org/research/cancerfactsstatistics/cancer-facts-figures-for-african-americans.*

10. *U.S. Department of Health and Human Services, Tobacco use among U.S. racial/ethnic minority groups — African Americans, American Indians and Alaska Natives, Asian Americans and Pacific Islanders, and Hispanics: A report of the surgeon general. , U.S. Department of Health and Human Services, Centers for Disease Control and Prevention, National Center for Chronic Disease Prevention and Health Promotion, Office on Smoking and Health.: Atlanta, Georgia*

11. *Murray, C.J. and Lopez, A.D., Alternative projections of mortality and disability by cause 1990-2020: global burden of disease study. The Lancet, 1997. 349: p. 1498-1504. PMID:*

12. *Critchley, J.A. and Capewell, S., Mortality risk reduction associated with smoking cessation in patients with coronary heart disease: a systematic review. Jama, 2003. 290(1): p. 86-97. PMID:*

13. *Fiore, M.C., Croyle, R.T., Curry, S.J., Cutler, C.M., Davis, R.M., Gordon, C., Healton, C., Koh, H.K., Orleans, C.T., Richling, D., Satcher, D., Seffrin, J., Williams, C., Williams, L.N., Keller, P.A., and Baker, T.B., Preventing 3 million premature deaths and helping 5*

million smokers quit: a national action plan for tobacco cessation. Am J Public Health, 2004. 94(2): p. 205-10. PMID:

14. *Trosclair, A., Husten, C., Pederson, L., and Dhillon, I., Cigarette smoking among adults – United States, 2000. Morbidity and Mortality Weekly Report, 2002. 51(29): p. 642-645. PMID:*
15. *Fiore, C., Jaen, C.R., and Baker, T.B., Treating Tobacco Use And Dependence: 2008 update. 2008, U.S. Department of Health and Human Services. Public Health Service: Rockville, MD*
16. *Centers for Disease Control and Prevention, O.o.S.a.H. Smoking & Tobacco Use. 2015 April 15, 2015 [cited 2015 April 27]; Available from: http://www.cdc.gov/tobacco/data_statistics/fact_sheets/fast_facts/.*
17. *Ekpu, V.U. and Brown, A.K., The Economic Impact of Smoking and of Reducing Smoking Prevalence: Review of Evidence. Tob Use Insights, 2015. 8: p. 1-35. PMID:*
18. *NIH State-of-the-Science Conference Statement on Tobacco Use: Prevention, Cessation, and Control. NIH Consens State Sci Statements, 2006. 23(3): p. 1-26. PMID:*
19. *Shahab, L. and McEwen, A., Online support for smoking cessation: a systematic review of the literature. Addiction, 2009. 104(11): p. 1792-804. PMID:*
20. *Myung, S.K., McDonnell, D.D., Kazinets, G., Seo, H.G., and Moskowitz, J.M., Effects of Web- and computer-based smoking cessation programs: meta-analysis of randomized controlled trials. Arch Intern Med, 2009. 169(10): p. 929-37. PMID:*
21. *Civljak, M., Sheikh, A., Stead, L.F., and Car, J., Internet-based interventions for smoking cessation. Cochrane Database Syst Rev, 2010(9): p. CD007078. PMID:*
22. *Lancaster, T. and Stead, L.F., Self-help interventions for smoking cessation. Cochrane Database Syst Rev, 2005(3): p. CD001118. PMID:*
23. *Sciamanna, C.N., Ford, D.E., Flynn, J.A., and Langford, C., An evidence-based interactive computer program to assist physicians in counseling smokers to quit. MD Computing, 1999. 16(5): p. 54-60. PMID:*
24. *Strecher, V.J., Computer-tailored smoking cessation materials: a review and discussion. Patient Education and Counseling, 1999. 36: p. 107-117. PMID:*
25. *Bental, D.S., Cawsey, A., and Jones, R., Patient information systems that tailor to the individual. Patient Education and Counseling, 1999. 36: p. 171-180. PMID:*
26. *Ramelson, H.Z., Friedman, R.H., and Ockene, J.K., An automated telephone-based smoking cessation education and counseling system. Patient Education and Counseling, 1999. 36: p. 131-144. PMID:*
27. *Lubalin, J., Consumer Health Informatics and Patient Decision-Making. 1997, Rockville MD: U.S. Department of Health and Human Services, U.S. Government Printing Office.*
28. *Ossip-Klein, D.J. and McIntosh, S., Quitlines in North America: evidence base and applications. Am J Med Sci, 2003. 326(4): p. 201-5. PMID:*
29. *HHS 'Quitline' helps Americans stop smoking. FDA Consum, 2005. 39(1): p. 4-5. PMID:*
30. *Sadasivam, R.S., Allison, J.J., Ray, M.N., Ford, D.E., and Houston, T.K., Using a Resource Effect Study Pre-Pilot to Inform a Large Randomized Trial: The Decide2Quit.Org Web-Assisted Tobacco Intervention (in press: AMIA-0373-A2012.R1). AMIA Annu Symp Proc, 2012. PMID:*
31. *PCORI Communication and Dissemination Research - Cycle 1. 2016 [cited 2016 May]; Available from: http://www.pcori.org/sites/default/files/PCORI-PFA-2016-Cycle-1-Communication-and-Dissemination-Research.pdf.*

32. *McCormack L, Sheridan S, Lewis M, Boudewyns V, Melvin CL, Kistler C, Lux LJ, Cullen K, and Lohr KN, Communication and Dissemination Strategies To Facilitate the Use of Health-Related Evidence, in Evidence Report/Technology Assessment, R.I.U.o.N.C.E.-b.P.C.u.C.N. 290-2007-10056-I, Editor. November 2013, Rockville, MD: Agency for Healthcare Research and Quality; No. 13(14)-E003-EF*

33. *Brownson, R.C., Colditz, G.A., and Proctor, E.K., Dissemination and implementation research in health : translating science to practice. 2012, Oxford ; New York: Oxford University Press. xxiii, 536 p.*

34. *Graham, A.L., Cobb, N.K., Papandonatos, G.D., Moreno, J.L., Kang, H., Tinkelman, D.G., Bock, B.C., Niaura, R.S., and Abrams, D.B., A randomized trial of Internet and telephone treatment for smoking cessation. Arch Intern Med, 2011. 171(1): p. 46-53. PMID:*

35. *Hughes, J.R. and Carpenter, M.J., The feasibility of smoking reduction: an update. Addiction (Abingdon, England), 2005. 100(8): p. 1074-1089. PMID:*

36. *Smit, E.S., Hoving, C., Schelleman-Offermans, K., West, R., and de Vries, H., Predictors of successful and unsuccessful quit attempts among smokers motivated to quit. Addict Behav, 2014. 39(9): p. 1318-24. PMID:*

37. *Glasgow, R.E., Klesges, L.M., Dzewaltowski, D.A., Estabrooks, P.A., and Vogt, T.M., Evaluating the impact of health promotion programs: using the RE-AIM framework to form summary measures for decision making involving complex issues. Health Educ Res, 2006. 21(5): p. 688-94. PMID:*

38. *Centers for Disease Control and Prevention (2011) Quitting Smoking Among Adults --- United States, 2001--2010. Morbidity and Mortality Weekly Report (MMWR) 60, 1513-1519.*

39. *Pew Research Center. Broadband vs. Dial-up Adoption Over Time. 2014 [cited 2015 October 10]; Available from: http://www.pewinternet.org/data-trend/internet-use/connection-type/.*

40. *Pew Research Center. Internet Use Over Time. 2014 [cited 2015 October 10]; Available from: http://www.pewinternet.org/data-trend/internet-use/internet-use-over-time/.*

41. *Pew Research Center's Internet and American Life Project. Chapter Two: Usage and Attitudes Toward Smartphones. 2015 [cited May 2016]; Available from: http://www.pewinternet.org/2015/04/01/chapter-two-usage-and-attitudes-toward-smartphones/.*

42. *Pew Research Center's Internet and American Life Project. Social Media Fact Sheet. 2014 [cited May 2016]; Available from: http://www.pewinternet.org/fact-sheets/social-networking-fact-sheet/.*

43. *Huang, G.C., Unger, J.B., Soto, D., Fujimoto, K., Pentz, M.A., Jordan-Marsh, M., and Valente, T.W., Peer influences: The impact of online and offline friendship networks on adolescent smoking and alcohol use. Journal of Adolescent Health, 2013. PMID:*

44. *Christakis, N.A. and Fowler, J.H., The collective dynamics of smoking in a large social network. New England journal of medicine, 2008. 358(21): p. 2249-2258. PMID:*

45. *Swider, S.M., Outcome effectiveness of community health workers: an integrative literature review. Public Health Nurs, 2002. 19(1): p. 11-20. PMID:*

46. *Eng, E., Parker, E., and Harlan, C., Lay health advisor intervention strategies: a continuum from natural helping to paraprofessional helping. Health Educ Behav, 1997. 24(4): p. 413-7. PMID:*
47. *Carroll, B., The hidden power of your customers : four keys to growing your business through existing customers. 2011, Hoboken, N.J.: Wiley. xvi, 240 p.*
48. *Marlin, B.M., Adams, R.J., Sadasivam, R., and Houston, T.K., Towards collaborative filtering recommender systems for tailored health communications. AMIA Annu Symp Proc, 2013. 2013: p. 1600-7. PMID:*
49. *Sadasivam, R.S., Cutrona, S.L., Volz, E., Rao, S.R., and Houston, T.K., Web-based peer-driven chain referrals for smoking cessation. Stud Health Technol Inform, 2013. 192: p. 357-61. PMID:*
50. *Wuyts, S., The connected customer : the changing nature of consumer and business markets. 2010, New York: Routledge. xviii, 353 p.*
51. *Sadasivam, R.S., Volz, E.M., Kinney, R.L., Rao, S.R., and Houston, T.K., Share2Quit: Web-Based Peer-Driven Referrals for Smoking Cessation. JMIR Res Protoc, 2013. 2(2): p. e37. PMID:*
52. *Sadasivam, R.S., Kinney, R.L., Delaughter, K., Rao, S.R., Williams, J.H., Coley, H.L., Ray, M.N., Gilbert, G.H., Allison, J.J., Ford, D.E., and Houston, T.K., Who participates in Web-assisted tobacco interventions? The QUIT-PRIMO and National Dental Practice-Based Research Network Hi-Quit studies. J Med Internet Res, 2013. 15(5): p. e77. PMID:*
53. *Kreuter, M., Tailoring health messages : customizing communication with computer technology. LEA's communication series. 2000, Mahwah, N.J.: L. Erlbaum. xiii, 270 p.*
54. *DiClemente, C.C., Prochaska, J.O., Fairhurst, S.K., Velicer, W.F., Velasquez, M.M., and Rossi, J.S., The process of smoking cessation: an analysis of precontemplation, contemplation, and preparation stages of change. J Consult Clin Psychol, 1991. 59(2): p. 295-304. PMID:*
55. *Bartholomew, L.K., Planning health promotion programs : an intervention mapping approach. 3rd ed. 2011, San Francisco, CA: Jossey-Bass. xx, 745 p.*
56. *Bandura, A., Social Foundations of Thought and Action: a social cognitive theory. 1986, Englewood Cliffs, NJ: Prentice Hall.*
57. *Shiffman, S., Paty, J.A., Rohay, J.M., Di Marino, M.E., and Gitchell, J., The efficacy of computer-tailored smoking cessation material as a supplement to nicotine polacrilex gum therapy. Arch Intern Med, 2000. 160(11): p. 1675-81. PMID:*
58. *Strecher, V.J., Computer-tailored smoking cessation materials: a review and discussion. Patient Educ Couns, 1999. 36(2): p. 107-17. PMID:*
59. *Brug, J., Steenhuis, I., van Assema, P., and de Vries, H., The impact of a computer-tailored nutrition intervention. Prev Med, 1996. 25(3): p. 236-42. PMID:*
60. *Kreuter, M.W. and Strecher, V.J., Do tailored behavior change messages enhance the effectiveness of health risk appraisal? Results from a randomized trial. Health Educ Res, 1996. 11(1): p. 97-105. PMID:*
61. *Kreuter, M.W., Community health promotion ideas that work : a field-book for practitioners. Jones and Bartlett series in health. 1998, Sudbury, Mass.: Jones and Bartlett Publishers. xviii, 206 p.*
62. *Segaran, T., Programming collective intelligence : building smart web 2.0 applications. 1st ed. 2007, Beijing ; Sebastapol CA: O'Reilly. xxi, 334 p.*

63. *Tom Vanderbilt. The Science Behind the Netflix Algorithms That Decide What You'll Watch Next. 2013 [cited 2014 August]; Available from: http://www.wired.com/2013/08/qq_netflix-algorithm/.*

64. *Joseph A. Konstan and Riedl, J. Deconstructing Recommender Systems: How Amazon and Netflix predict your preferences and prod you to purchase. 2012 24 Sep [cited 2014 August]; Available from: http://spectrum.ieee.org/computing/software/deconstructing-recommender-systems.*

65. *Sadasivam, R.S., Cutrona, S.L., Kinney, R.L., Marlin, B.M., Mazor, K.M., Lemon, S.C., and Houston, T.K., Collective-Intelligence Recommender Systems: Advancing Computer Tailoring for Health Behavior Change Into the 21st Century. J Med Internet Res, 2016. 18(3): p. e42. PMID:*

66. *Ryan, R.M. and Deci, E.L., Self-determination theory and the facilitation of intrinsic motivation, social development, and well-being. The American psychologist, 2000. 55(1): p. 68-78. PMID:*

67. *Deci, E.L. and Ryan, R.M., The "what" and "why" of goal pursuits: Human needs and the self-determination of behavior. Psychological Inquiry, 2000. 11: p. 227-268. PMID:*

68. *Vallerand, R.J., Deci and Ryan's self-determination theory: A view from the hierarchical model of intrinsic and extrinsic motivation Psychological Inquiry, 2000. 11(4): p. 312-318. PMID:*

69. *Flynn, B.S., Worden, J.K., Bunn, J.Y., Connolly, S.W., and Dorwaldt, A.L., Evaluation of smoking prevention television messages based on the elaboration likelihood model. Health Educ Res, 2011. PMID:*

70. *Grant, A., Berlin, A., and Freeman, G.K., The impact of a student learning journal: a two-stage evaluation using the Nominal Group Technique. Med Teach, 2003. 25(6): p. 659-61. PMID:*

71. *Miller, D., Shewchuk, R., Elliot, T.R., and Richards, S., Nominal group technique: a process for identifying diabetes self-care issues among patients and caregivers. Diabetes Educ, 2000. 26(2): p. 305-10, 312, 314. PMID:*

72. *Sadasivam, R.S., Delaughter, K., Crenshaw, K., Sobko, H.J., Williams, J.H., Coley, H.L., Ray, M.N., Ford, D.E., Allison, J.J., and Houston, T.K., Development of an interactive, Web-delivered system to increase provider-patient engagement in smoking cessation. J Med Internet Res, 2011. 13(4): p. e87. PMID:*

73. *Coley, H.L., Sadasivam, R.S., Williams, J.H., Volkman, J.E., Schoenberger, Y.M., Kohler, C.L., Sobko, H., Ray, M.N., Allison, J.J., Ford, D.E., Gilbert, G.H., Houston, T.K., National Dental, P., and Group, Q.C., Crowdsourced peer- versus expert-written smoking-cessation messages. Am J Prev Med, 2013. 45(5): p. 543-50. PMID:*

74. *Fiore, M., Jaen, C., Baker, T., Bailey, W., Benowitz, N., Curry, S., Dorfman, S., Froelicher, E., Goldstein, M., Healton, C., Henderson, P., Heyman, R., Koh, H., Kottke, T., Lando, H., Mecklenburg, R., Mermelstein, R., Mullen, P., Orleans, C., Robinson, L., Stitzer, M., Tommasello, A., Villejo, L., and Wewers, M., Treating Tobacco Use and Dependence: 2008 Update. Clinical Practice Guideline. May2008, Rockville, MD: U.S. Department of Health and Human Services. Public Health Service.*

75. *Bandura, A., Social Foundations of Thought and Action: a social cognitive theory. 1986, Englewood Cliffs, NJ: Prentice Hall.*

76. Melville, P., Mooney, R.J., and Nagarajan, R., Content-Boosted Collaborative Filtering for Improved Recommendations, in Proceedings of the Eighteenth National Conference on Artificial Intelligence (AAAI-02). 2002: Edmonton, Alberta. p. 187-192
77. Adams, R.J., Sadasivam, R.S., Balakrishnan, K., Kinney, R.L., Houston, T.K., and Marlin, B.M., PERSPeCT: collaborative filtering for tailored health communications, in RecSys'14. 2014: Foster City, Silicon Valley, CA. p. 329-332.10.1145/2645710.2645768
78. Sadasivam, R.S., Allison, J.J., Ray, M.N., Ford, D.E., and Houston, T.K., Using a resource effect study pre-pilot to inform a large randomized trial: the Decide2Quit.Org Web-assisted tobacco intervention. AMIA Annu Symp Proc, 2012. 2012: p. 789-98. PMID:
79. Houston, T.K., Sands, D.Z., Jenckes, M.W., and Ford, D.E., Experiences of patients who were early adopters of electronic communication with their physician: satisfaction, benefits, and concerns. Am J Manag Care, 2004. 10(9): p. 601-8. PMID:
80. Sciamanna, C.N., Novak, S.P., Houston, T.K., Gramling, R., and Marcus, B.H., Visit satisfaction and tailored health behavior communications in primary care. Am J Prev Med, 2004. 26(5): p. 426-30. PMID:
81. Centers for Disease Control and Prevention (CDC). 2010 Behavioral Risk Factor Surveillance System Survey Questionnaire. Available from: http://www.cdc.gov/brfss/pubs/index.htm#users. Archived at http://www.webcitation.org/6BGROyW6b.
82. Jarvis MJ, T.-P.H., Feyerabend C, Vesey C, Saloojee Y, Comparison of tests used to distinguish smokers from non-smokers. American Journal of Public Health, 1987. 77(11): p. 1435-38. PMID:
83. Gaalema, D.E., Higgins, S.T., Bradstreet, M.P., Heil, S.H., and Bernstein, I.M., Using NicAlert strips to verify smoking status among pregnant cigarette smokers. Drug Alcohol Depend, 2011. 119(1-2): p. 130-3. PMID:
84. Marrone, G.F., Paulpillai, M., Evans, R.J., Singleton, E.G., and Heishman, S.J., Breath carbon monoxide and semiquantitative saliva cotinine as biomarkers for smoking. Hum Psychopharmacol, 2010. 25(1): p. 80-3. PMID:
85. Raja, M., Garg, A., Yadav, P., Jha, K., and Handa, S., Diagnostic Methods for Detection of Cotinine Level in Tobacco Users: A Review. J Clin Diagn Res, 2016. 10(3): p. ZE04-6. PMID:
86. Rabe-Hesketh, S., Skrondal, A., and Pickles, A., Reliable estimation of generalized linear mixed models using adaptive quadrature. Stata Journal, 2002. 2(1): p. 1-21. PMID:
87. Velicer, W.F., Prochaska, J.O., Rossi, J.S., and Snow, M.G., Assessing outcome in smoking cessation studies. Psychol Bull, 1992. 111(1): p. 23-41. PMID:
88. Cooke, F., Bullen, C., Whittaker, R., McRobbie, H., Chen, M.H., and Walker, N., Diagnostic accuracy of NicAlert cotinine test strips in saliva for verifying smoking status. Nicotine Tob Res, 2008. 10(4): p. 607-12. PMID:
89. Hintze, J., NCSS and PASS. Number Cruncher Statistical Systems, NCSS, LLC.: Kaysville, UT.
90. Whitehead, J., Sample size calculations for ordered categorical data. Stat Med, 1993. 12(24): p. 2257-71. PMID:
91. Carpenter, M.J., Hughes, J.R., Gray, K.M., Wahlquist, A.E., Saladin, M.E., and Alberg, A.J., Nicotine therapy sampling to induce quit attempts among smokers unmotivated to quit: a randomized clinical trial. Arch Intern Med, 2011. 171(21): p. 1901-7. PMID:

92. Austin, P.C., Manca, A., Zwarenstein, M., Juurlink, D.N., and Stanbrook, M.B., A substantial and confusing variation exists in handling of baseline covariates in randomized controlled trials: a review of trials published in leading medical journals. J Clin Epidemiol, 2010. 63(2): p. 142-53. PMID:

93. Senn, S., Testing for baseline balance in clinical trials. Stat Med, 1994. 13(17): p. 1715-26. PMID:

94. Ray, M.N., Allison, J.J., Coley, H.L., Williams, J.H., Kohler, C., Gilbert, G.H., Richman, J.S., Kiefe, C.I., Sadasivam, R.S., Houston, T.K., and National, D.C.G., Variations in tobacco control in National Dental PBRN practices: the role of patient and practice factors. Spec Care Dentist, 2013. 33(6): p. 286-93. PMID:

95. Sadasivam, R.S., Hogan, T.P., Volkman, J.E., Smith, B.M., Coley, H.L., Williams, J.H., Delaughter, K., Ray, M.N., Gilbert, G.H., Ford, D.E., Allison, J.J., Houston, T.K., National Dental, P., and Groups, Q.C., Implementing point of care "e-referrals" in 137 clinics to increase access to a quit smoking internet system: the Quit-Primo and National Dental PBRN HI-QUIT Studies. Transl Behav Med, 2013. 3(4): p. 370-8. PMID:

96. Rocheleau, M., Sadasivam, R.S., Baquis, K., Stahl, H., Kinney, R.L., Pagoto, S.L., and Houston, T.K., An observational study of social and emotional support in smoking cessation Twitter accounts: content analysis of tweets. J Med Internet Res, 2015. 17(1): p. e18. PMID:

97. Chen, P.W. When Patients Share Their Stories, Health May Improve. 2011 11/13/2013]; Available from: http://www.nytimes.com/2011/02/10/health/views/10chen.html?_r=3&ref=health&.

98. Weintraub, K. Talk therapy: Storytelling may help what ails her - just as her story has helped others. 2011 11/13/2013]; Available from: http://www.boston.com/lifestyle/health/articles/2011/03/21/patients_benefit_from_listening_to_the_stories_of_those_dealing_with_similar_ailments/.

99. Park, A. Storytelling May Help Lower Blood Pressure. 2011 11/13/2013]; Available from: http://healthland.time.com/2011/01/17/story-telling-may-help-to-lower-blood-pressure/.

100. Gopinath, B., Baur, L.A., Hardy, L.L., Kifley, A., Rose, K.A., Wong, T.Y., and Mitchell, P., Relationship between a range of sedentary behaviours and blood pressure during early adolescence. J Hum Hypertens, 2012. 26(6): p. 350-6. PMID:
