# Supplementary material for: Effect of a Machine Learning Recommender System and Viral Peer Marketing Intervention on Smoking Cessation: A Randomized Clinical Trial
Source: JAMA Netw Open. 2023 Jan 12;6(1):e2250665. doi: 10.1001/jamanetworkopen.2022.50665 (PMC9856644; doi:10.1001/jamanetworkopen.2022.50665)
Supplement: Supplement 2. — eTable 1. Comparison of Baseline Characteristics Between Individuals With and Without Viral Tool Kit eTable 2. Comparison of Baseline Characteristics Between Individuals Randomized to ML Recommender and Standard Messaging eTable 3. Smoking-Cessation Rates at 6 Months for Each 3 Hypotheses With Missing Outcomes Treated as Smoking eTable 4. Smoking-Cessation Rates at 6 mo for 3 Hypotheses With Missing Outcomes Treated Using Multiple Imputation [file jamanetwopen-e2250665-s002.pdf]

Supplemental Online Content

Faro JM, Chen J, Flahive J, et al. Effect of a machine learning recommender system and viral peer marketing intervention on smoking cessation: a randomized clinical trial. *JAMA Netw Open.* 2023;6(1):e2250665. doi:10.1001/jamanetworkopen.2022.50665

eTable 1. Comparison of Baseline Characteristics Between Individuals With and Without Viral Tool Kit

eTable 2. Comparison of Baseline Characteristics Between Individuals Randomized to ML Recommender and Standard Messaging

eTable 3. Smoking-Cessation Rates at 6 Months for Each 3 Hypotheses With Missing Outcomes Treated as Smoking

eTable 4. Smoking-Cessation Rates at 6 mo for 3 Hypotheses With Missing Outcomes Treated Using Multiple Imputation

This supplemental material has been provided by the authors to give readers additional information about their work.

© 2023 Faro JM et al. *JAMA Network Open.*

eTable 1. Comparison of Baseline Characteristics Between Individuals With and Without Viral Tool Kit

**Variable**	**ViralToolkit**	**NoViralToolkit**	***P* value**
	(n=745)	(n=742)	
	n (%)	n (%)	
Age			0.12
19-34 years	236 (31.7%)	208 (28.0%)	
35-54 years	259 (34.8%)	249 (33.6%)	
55+ years	250 (33.6%)	285 (38.4%)	
Gender			0.007*
Female	529 (71.0%)	572 (77.1%)	
Male	216 (29.0%)	170 (22.9%)	
Race			0.98
African-American/Black	95 (13.3%)	93 (13.1%)	
White	583 (81.9%)	584 (82.3%)	
Other[a]	34 (4.8%)	33 (4.6%)	
Ethnicity			0.40
Not Hispanic or Latino	652 (93.0%)	639 (91.8%)	
Hispanic or Latino	49 (7.0%)	57 (8.2%)	
Education			0.018[b]
High school or less education	168 (34.2%)	131 (26.4%)	
Some college or technical school	206 (42.0%)	220 (44.4%)	
College graduate	117 (23.8%)	145 (29.2%)	
How hard it is for you/family to pay for medical care			0.43
Very hard or hard	172 (36.0%)	158 (32.5%)	
Somewhat hard	165 (34.5%)	185 (38.1%)	
Not very hard	141 (29.5%)	143 (29.4%)	
Readiness to Quit Smoking			0.46
I am not thinking about quitting	34 (4.6)	28 (3.8)	
I am thinking of quitting or I have set a quit date	597 (81)	585 (79)	
I quit today or I have already quit	109 (15)	123 (17)	
Number of Cigarettes Smoked per Day			0.06
0-5	108 (14.5%)	87 (11.7%)	
6-20	499 (67.1%)	486 (65.5%)	
21 or more	137 (18.4%)	169 (22.8%)	

MLRecommender=Machine learning recommender computer-tailored motivational texting intervention; Stdmessaging= standard motivational texting intervention; ViralToolkit=Peer recruitment toolkit
[a]Includes Asian, American Indian or Alaska Native, Native Hawaiian or Other Pacific Islander
[b]Indicates statistically significant (P<0.05)

© 2023 Faro JM et al. *JAMA Network Open.*

eTable 2. Comparison of Baseline Characteristics Between Individuals Randomized to ML Recommender and Standard Messaging

Variable	ML Recommender	Std Messaging	*P* value
	(n=745)	(n=742)	
	n (%)	n (%)	
Age			0.27
19-34 years	207 (27.9%)	237 (31.8%)	
35-54 years	261 (35.2%)	247 (33.1%)	
55+ years	273 (36.8%)	262 (35.1%)	
Gender			0.31
Female	540 (72.9%)	561 (75.2%)	
Male	201 (27.1%)	185 (24.8%)	
Race			0.15
African-American	98 (13.8%)	90 (12.7%)	
White	588 (82.6%)	579 (81.5%)	
Other	26 (3.7%)	41 (5.8%)	
Ethnicity			0.06
Not Hispanic or Latino	659 (93.7%)	632 (91.1%)	
Hispanic or Latino	44 (6.3%)	62 (8.9%)	
Education			0.31
High school or less education	142 (28.3%)	157 (32.4%)	
Some college or technical school	219 (43.6%)	207 (42.7%)	
College graduate	141 (28.1%)	121 (24.9%)	
How hard it is for you/family to pay for medical care			0.12
Very hard or hard	173 (35.3%)	157 (33.1%)	
Somewhat hard	163 (33.3%)	187 (39.5%)	
Not very hard	154 (31.4%)	130 (27.4%)	
Readiness to Quit Smoking			0.15
I am not thinking about quitting	27 (3.7%)	35 (4.7%)	
I am thinking of quitting or I have set a quit date	602 (82.1%)	580 (78.1%)	
I quit today or I have already quit	104 (14.2%)	128 (17.2%)	
Number of Cigarettes Smoked per Day			0.54
0-5	92 (12.4%)	103 (13.8%)	
6-20	501 (67.6%)	484 (65.0%)	
21 or more	148 (20.0%)	158 (21.2%)	

MLRecommender=Machine learning recommender computer-tailored motivational texting intervention; Stdmessaging= standard motivational texting intervention; ViralToolkit=Peer recruitment toolkit
* Indicates statistically significant (P<0.05).

© 2023 Faro JM et al. *JAMA Network Open.*

eTable 3. Smoking-Cessation Rates at 6 Months for Each 3 Hypotheses With Missing Outcomes Treated as Smoking

	n/N (%)	Model unadjusted		Model adjusted for covariates [a]	
		Odds Ratio (95% Confidence Interval)	*P* value	Odds Ratio (95% Confidence Interval)	*P* value
**Hypothesis H1**					
**Sub-hypothesis 1: Group A vs. Group B**					
Fully enhanced	89/371 (24)	1.73 (1.20, 2.51)	0.004*	1.79 (1.17, 2.72)	0.007*
MLRecommender and NoViralToolkit	57/370 (15)	Reference		Reference	
**Sub-hypothesis 2: Group A vs. Group C**					
Fully enhanced	89/371 (24)	1.03 (0.73, 1.44)	0.88	0.96 (0.65, 1.42)	0.83
StdMessaging and ViralToolkit	88/374 (24)	Reference		Reference	
**Sub-hypothesis 3: Group A vs. Group D**					
Fully enhanced	89/371 (24)	1.41 (0.99, 2.01)	0.06	1.19 (0.79, 1.78)	0.40
StdMessaging and NoViralToolkit	68/372 (18)	Reference		Reference	
**Hypothesis 2**					
**Groups (A and C) vs. Groups (B and D)**					
ViralToolkit	125/742 (17)	1.54 (1.19, 1.99)	0.001*	1.48 (1.11, 1.98)	0.01*
NoVitalToolkit	177/745 (24)	Reference		Reference	
**Hypothesis 3**					
**Groups (A and B) versus Groups (C and D)**					
MLRecommender (A+B)	146/741 (20)	0.93 (0.72, 1.20)	0.56	0.81 (0.61, 1.08)	0.16
StdMessaging (C+D)	156/746 (21)	Reference		Reference	

MLRecommender=Machine learning recommender computer-tailored motivational texting intervention; Stdmessaging= standard motivational texting intervention; ViralToolkit=Peer recruitment toolkit
Group A – Fully Enhanced Group – MLRecommender and ViralToolkit
Group B – MLRecommender with NoViralToolkit
Group C – StdMessaging with ViralToolkit
Group D – StdMessaging with NoViralToolkit
[a] Multivariable logistic regression was adjusted for sex and education level.
*Indicates statistically significant (P<0.05).

© 2023 Faro JM et al. *JAMA Network Open.*

eTable 4. Smoking-Cessation Rates at 6 mo for 3 Hypotheses With Missing Outcomes Treated Using Multiple Imputation

	n/N (%)	Model unadjusted		Model adjusted for covariates [a]	
		**Odds Ratio (95% Confidence Interval)**	***P* value**	**Odds Ratio (95% Confidence Interval)**	***P* value**
**Hypothesis 1**					
**Sub-hypothesis 1: Group A vs. Group B**					
Fully enhanced				1.77 (1.16, 2.70)	0.01*
MLRecommender and NoViralToolkit		Reference		Reference	
**Sub-hypothesis 2: Group A vs. Group C**					
Fully enhanced				0.90 (0.58, 1.38)	0.63
StdMessaging and ViralToolkit		Reference		Reference	
**Fully enhanced vs. Standard (Hypothesis 1)**					
Fully enhanced				1.44 (0.95, 2.19)	0.09
StdMessaging and NoViralToolkit		Reference		Reference	
**Hypothesis 2**					
**Groups (A and C) vs. Groups (B and D)**					
ViralToolkit				1.68 (1.24, 2.28)	0.001*
No ViralToolkit		Reference		Reference	
**Hypothesis 3**					
**Groups (A and B) versus Groups (C and D)**					
MLRecommender (A+B)				0.86 (0.63, 1.18)	0.35
StdMessaging (C+D)		Reference		Reference	

Group A – Fully Enhanced Group – MLRecommender and ViralToolkit
Group B – MLRecommender with NoViralToolkit
Group C – StdMessaging with ViralToolkit
Group D – StdMessaging with NoViralToolkit
[a] Multivariable logistic regression was adjusted for age, race, sex, education level, and smoking status and number of cigarettes per day measured at baseline.
* Indicates statistically significant (P<0.05).

© 2023 Faro JM et al. *JAMA Network Open.*
