# Supplementary material for: Effect of a Machine Learning Recommender System and Viral Peer Marketing Intervention on Smoking Cessation: A Randomized Clinical Trial
Source: JAMA Netw Open. 2023 Jan 12;6(1):e2250665. doi: 10.1001/jamanetworkopen.2022.50665 (PMC9856644; doi:10.1001/jamanetworkopen.2022.50665)
Supplement: Supplement 3. — Data Sharing Statement [file jamanetwopen-e2250665-s003.pdf]

Data Sharing Statement

Faro JM, Chen J, Flahive J, et al. Effect of a machine learning recommender system and viral peer marketing intervention on smoking cessation: a randomized clinical trial. *JAMA Netw Open*. 2023;6(1):e2250665. doi:10.1001/jamanetworkopen.2022.50665

Data

Data available: Yes
Data types: Deidentified participant data
How to access data: Rajani.sadasivam@umassmed.edu **When available:** With publication

Supporting Documents

Document types: None

Additional Information

Who can access the data: Anyone requesting the data.
Types of analyses: For any purpose.
Mechanisms of data availability: With a signed data access agreement.

© 2023 Faro JM et al. *JAMA Network Open*.
